# Nonlinear adaptive NeuroFuzzy feedback linearization based MPPT control schemes for photovoltaic system in microgrid

**DOI:** 10.1371/journal.pone.0234992

**Published:** 2020-06-30

**Authors:** Muhammad Awais, Laiq Khan, Saghir Ahmad, Sidra Mumtaz, Rabiah Badar

**Affiliations:** Department of Electrical and Computer Engineering, COMSATS University Islamabad, Abbottabad, Pakistan; Huazhong University of Science and Technology, CHINA

## Abstract

Renewable energy resources connected to a single utility grid system require highly nonlinear control algorithms to maintain efficient operation concerning power output and stability under varying operating conditions. This research work presents a comparative analysis of different adaptive Feedback Linearization (FBL) embedded Full Recurrent Adaptive NeuroFuzzy (FRANF) control schemes for maximum power point tracking (MPPT) of PV subsystem tied to a smart microgrid hybrid power system (SMG-HPS). The proposed schemes are differentiated based on structure and mathematical functions used in FRANF embedded in the FBL model. The comparative analysis is carried out based on efficiency and performance indexes obtained using the power error between the reference and the tracked power for three cases; a) step change in solar irradiation and temperature, b) partial shading condition (PSC), and c) daily field data. The proposed schemes offer enhanced convergence compared to existing techniques in terms of complexity and stability. The overall performance of all the proposed schemes is evaluated by a spider chart of multivariate comparable parameters. Adaptive PID is used for the comparison of results produced by proposed control schemes. The performance of Mexican hat wavelet-based FRANF embedded FBL is superior to the other proposed schemes as well as to aPID based MPPT scheme. However, all proposed schemes produce better results as compared to conventional MPPT control in all cases. Matlab/Simulink is used to carry out the simulations.

## Introduction

The energy demand of the globe is mainly fulfilled by fossil fuel. Increasing energy demand and limitation of fossil fuel supplies boost the cost of electricity. The environmental hazards due to greenhouse emission and scarcity of fossil fuel supplies diverted the focus towards renewable energy resources. Renewable power is clean, sustainable, green, economical, and durable. The productivity of renewable energy resources depends on meteorological conditions. Thus, a single stand-alone source is unable to supply continuous reliable energy. Therefore several renewable and non-renewable resources are integrated to form a single HPS. Solar energy is a huge reservoir of green energy blessed to this planet [1, 2]. To utilize it most reliably and efficiently needs its conversion into electrical energy [3]. Many researchers struggle for the extraction of maximum power from photovoltaic (PV) arrays as its demand increases in both stand-alone and grid-connected modes [4]. The grid-connected mode is trending these days due to drawbacks like high cost, heavy batteries, and regular maintenance related to traditional stand-alone PV systems [5, 6].

Different topologies, circuits, and control algorithms are required for the extraction of maximum power from PV. Maximum power tracking from a PV system is of interest for a long time and is usually preferred using a boost converter due to its advantages over other techniques [7, 8]. *P* − *V* and *I* − *V* characteristics of the PV system vary according to the atmospheric conditions, thus maximum power tracking becomes difficult [5, 8, 9]. Numerous MPPT techniques are practiced and proposed for the extraction of maximum power from the PV system that is then supplied to the grid through inverters [10].

Literature studies show that various conventional and intelligent methods are used for the MPPT of the PV system. The conventional techniques include perturb and observe (P&O) [11–14], fractional open-circuit voltage (OCV) [15], Hill Climbing algorithm (HCA) [16], incremental conductance (IC) [17], and ripple correlation (RC) [18] method. P&O, IC, and HCA have the drawback of oscillations around maximum power point (MPP), thus loses power and also show degraded energy conversion efficiency. Under fast varying conditions, P&O and IC have a slow response. RC algorithm needs prior knowledge of the phase relationship between the DC power ripple and DC voltage, to determine the MPP. Also, the implementation of the RC method is quite complex. The accuracy and efficiency of both OCV and SCC are low.

Advanced MPPT control techniques include artificial neural network (ANN) [19], fuzzy logic control (FLC) [20], feedback linearization [21], adaptive NeuroFuzzy [22], wavelet-based NeuroFuzzy [8, 23], and adaptive feedback linearization based NeuroFuzzy [9]. The major drawback of FLC is the exponential growth in the number of membership functions and the fuzzy if-then rules with an increase in variables. The ANN model requires periodic training to ensure convergence to the accurate MPP. Feedback linearization is a recent technique used for the MPPT problem [5, 9]. This technique decouples a nonlinear system into linear subsystems, thus linear control laws can be implemented on these decoupled subsystems. Feedback linearization has its applications in the control of robotics [24, 25], HVDC link [26], motor control [27], etc. However, for un-modeled system dynamics, the classical feedback linearization control has limited robustness and stability [25]. Adaptive feedback linearization-based NeuroFuzzy algorithms are used to nullify the drawbacks of classical feedback linearization. The NeuroFuzzy algorithm combines the reasoning method of humans in fuzzy systems with the learning abilities of neural networks. This hybrid adaptive scheme can deal with system nonlinearities, uncertainties, and fluctuations. Therefore, in an adaptive feedback linearization-based NeuroFuzzy control scheme, the adaptive feedback linearization control is applied to the nonlinear system identified by the hybrid NeuroFuzzy inference systems. In [8, 9] and [23] NeruoFuzzy schemes are presented for the identification and control of grid-connected PV subsystems. A feedforward NeuroFuzzy scheme is presented having static mapping thus time-domain dynamical responses require a larger number of neurons [28]. It limits the number of inputs and can explore the characteristics of local structure [28–30]. On the other hand, recurrent NeuroFuzzy control schemes consist of feedback and feedforward connections between layers [28]. The neurons in this scheme form complicated dynamics and use their natural temporal operation to deal with time-varying input/output [28, 29].

Mismatching of PV cells along with the weather and environmental changes like shadows of buildings, dust, moving clouds, and trees give rise to partial shading conditions (PSC), which result in power loss and produce numerous local MPPs (LMPPs) [31–33]. Numerous conventional and non-conventional techniques were developed to tackle PSC in the near past but they have drawbacks of lager power fluctuation, lower power output, and complexity of control design in some cases [32–37].

This paper presents a comparison of the performance of four different adaptive feedback linearization (FBL) techniques incorporated with full recurrent adaptive NeuroFuzzy (FRANF) based controllers for a PV system in a grid-integrated SMG-HPS for three different cases.

Seven-layered full recurrent adaptive NeuroFuzzy structure embedded with four different mathematical functions and wavelets is used to estimate the nonlinear functions of FBL control. FRANF structures are based on Standard Additive Model, Fourier Series, Mexican Hat Wavelet, and Chebyshev Wavelet for the estimation purpose. An online learning algorithm based on the gradient-decent method is applied to update all the parameters of the FRANF structure for each proposed control scheme adaptively. Three different cases are used on the same system to test the performance of proposed controllers. The extraction of maximum power under varying conditions from the PV system in these scenarios is challenging. The comparison of the proposed control schemes is based on the power quality of the SMG-HPS as well as extracted power and performance indexes obtained during the simulation process. The performance of the proposed control schemes is evaluated against the adaptive PID (aPID) control scheme.

The article comprised of four major sections. Section 1 describes the testbed for this research work. Section 2 gives detail of the proposed control schemes for the PV subsystem. Section 3 and 4 provide details of results and conclusion respectively.

## 1 SMG-HPS and PV subsystem

The sources in the testbed for this research work consists of wind-turbine (WT), electrolyzer, micro-turbine (MT), solid oxide fuel cell (SOFC), PV, Micro-hydro (MH), Bio-mass (BM), super-capacitor (SC), batteries, and utility grid (UG). The loads are prosumer, residential load, charging station (CS), and plug-in hybrid-electric vehicles (PHEVs).

**Fig 1:**
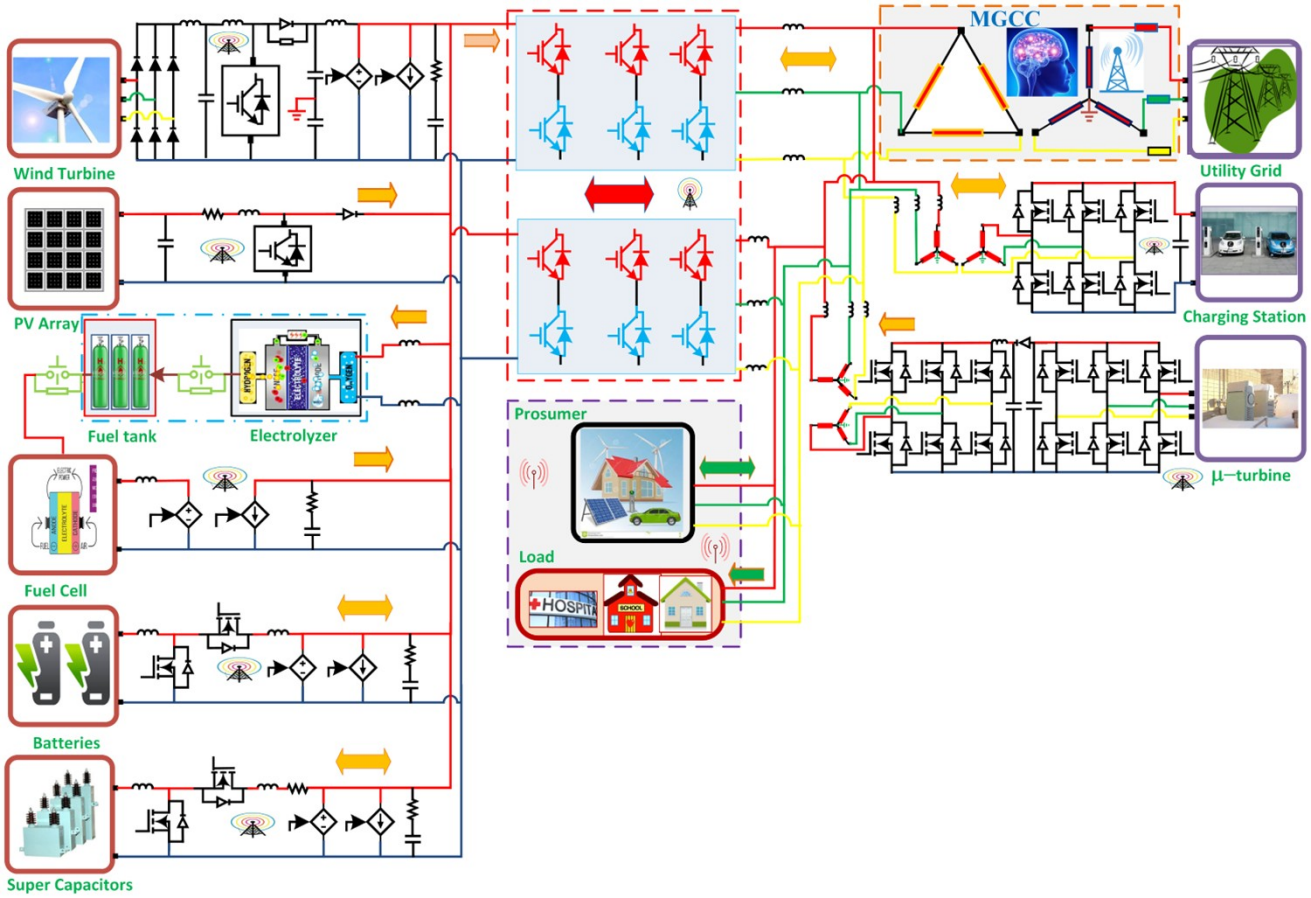
Smart micro-grid hybrid power system.

Renewable resources like WT, electrolyzer, PV, SOFC, SC, and batteries are connected to DC link and deliver power to the connected converters. These converters are connected to the AC bus that has UG, MT, MH, CS, BM, along with all types of loads attached to it as shown in Fig 1. Specifications of all the sources are described in section 3 Table 1. All the source converters except PV are being controlled by aPID.

**Table 1:** Parameters of the SMG system.

Source	Rating
UG	11 kV
PV	260 kW
WT	100 kW
Electrolyzer	150 kW
SOFC	260 kW
MT	200 kVA
SC	165 F
Batteries	200 Ah

### 1.1 PV cell model

A simple pn-junction that converts solar irradiation into electric energy is known as PV cell [32]. It is comprised of current source, parallel diode and a series resistor, which is further connected to boost converter [8, 32]. PV cells are combined in clusters which are then connected to one-another is series and parallel fashion to obtain the desired power level [9, 32, 33]. The relationship between output voltage and current of PV cell is given as: [6, 8, 19, 32, 33]

$$\begin{array}{ccc} i_{pv} & = & n_{p}i_{p}-n_{p}I_{D}(exp[\frac{q}{AKT_{c}}(\frac{v_{pv}}{n_{s}}+\frac{R_{s}i_{pv}}{n_{p}})]-1) \end{array}$$ (1)

where all the symbols are defined in Nomenclature. Photo-current, *i*_*p*_ can be determined by solar irradiation as:

$$\begin{array}{ccc} i_{p} & = & (i_{s}+k_{i}(T_{c}-Tref))\frac{Z}{1000} \end{array}$$ (2)

Beside this, saturation current, *i*_*s*_ of PV cell varies with temperature and can be related as:

$$\begin{array}{ccc} i_{s} & = & i_{RS}{\left[\frac{T_{c}}{T_{ref}}\right]}^{3}exp[\frac{qe_{g}}{Ak}(\frac{1}{T_{ref}}-\frac{1}{T_{c}})] \end{array}$$ (3)

The Eqs (1), (2) and (3) are used to design PV system and also show that the output of a PV array depends on solar irradiance and temperature of the environment [32].

## 2 Adaptive feedback linearization embedded NeuroFuzzy MPPT for PV system

### 2.1 Control law design

Feedback linearization is a tool that transforms nonlinear system dynamics into linear ones algebraically, either fully or partly, hence linear control techniques can be applied. nonlinearities can be eliminated from any nonlinear system represented in a companion form as [38]:

$$\begin{array}{c} y^{n}=f\left(x\right)+g\left(x\right)u_{MPPT} \end{array}$$ (4)

where *y* ∈ ℜ is plant output, *n* ∈ Z is relative degree of system, *f(x)* and *g(x)* are unknown nonlinear functions, *u*_*MPPT*_ ∈ ℜ is control input, and $x=\left[y,\dot{y},\ldots ,y^{n-1}\right){]}^{T}\;\in \;{ℜ}^{n}$ is the state-space vector. The control problem is to find *u*_*MPPT*_ that assures *y*(*t*) follows the desired trajectory *y*_*d*_(*t*). If a new input *v* represents the plant’s input then:

$$\begin{array}{c} u_{MPPT}=\frac{1}{g(x)}\left[-f\left(x\right)+v\right] \end{array}$$ (5)

Using the control law of Eq (5) in Eq (4), nonlinear terms will be canceled and input-output integral form is obtained as:

$$\begin{array}{cc} y^{n} & =v \end{array}$$ (6)

The nonlinear functions in Eq (5) are estimated using NeuroFuzzy systems. $\hat{f}\left(x\right)$ represents the estimated *f(x)* and $\hat{g}\left(x\right)$ represents *g(x)*. The adaptive feedback control law can be rewritten as:

$$\begin{array}{c} u_{MPPT}=\frac{1}{\hat{g}\left(x\right)}\left[-\hat{f}\left(x\right)+v\right] \end{array}$$ (7)

The control law of Eq (7) is based on control input *v* = −*K*_*v*_℘ − *Y*_*D*_ and identified nonlinear functions. where *K*_*v*_ is constant and ℘ the tracking error is given as [38]:

$$\begin{array}{c} {℘}^{T}=[\wedge^{T}\;\;\;1]e \end{array}$$ (8)

where ∧ = [λ_1_ λ_2_ ⋯  λ_*n*-1_] is constant weight vector and *e* = *x* − *x*_*d*_ represents error matrix.

Online adoption of ∧ ensures occupation of poles of *s*^*n*−1^ + λ_*n*−1_*s*^*n*−2^ + ⋯ + λ_1_ in left half of complex plane. To bring the tracking error to zero, the following control law is entertained by identification of $\hat{f}\left(x\right)$ and $\hat{g}\left(x\right)$ through FRANF, where $\left[\hat{g}\left(x\right)>0\right]$ is obtained by using saturation block and identifier is set to give 0.1 as output if $\hat{g}\left(x\right)=0$ during identification.

$$\begin{array}{c} u_{MPPT}={\hat{g}}^{-1}\left(x\right)[-\hat{f}\left(x\right)+v] \end{array}$$ (9)

**Fig 2:**
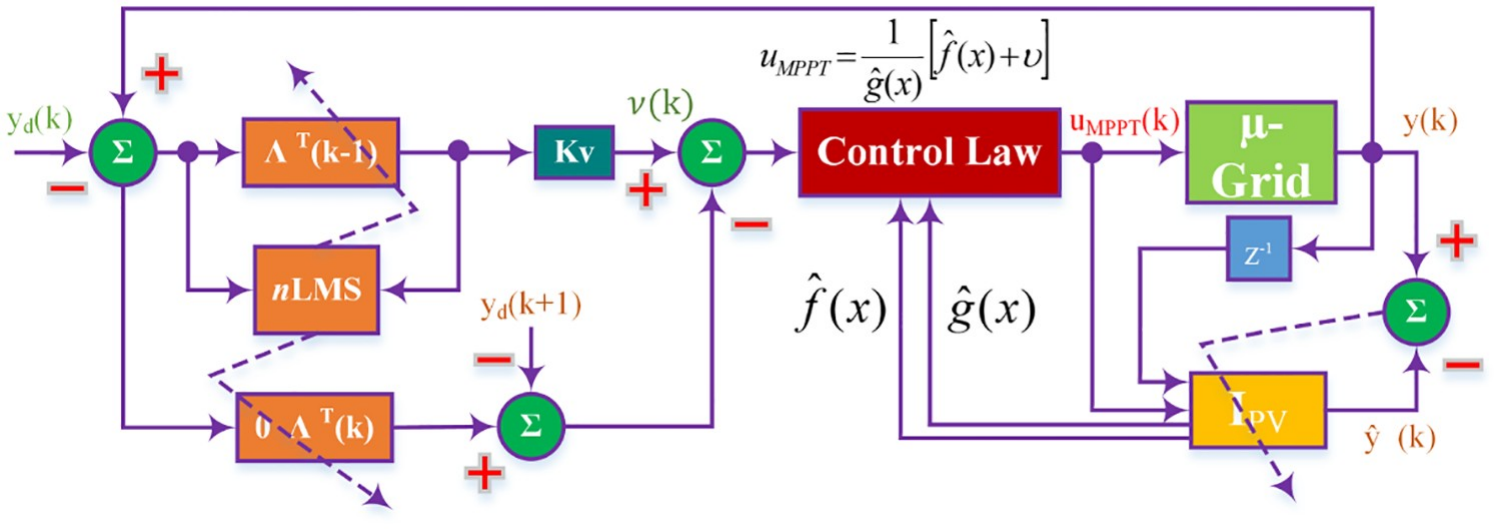
Feedback linearization based closed-loop system.

To generate appropriate control law, FBL control coefficients ∧ are updated using *n* LMS algorithm, as:

$$\begin{array}{c} {\hat{\wedge }}^{\left(i\right)}={\hat{\wedge }}^{\left(i-1\right)}+\frac{\varphi^{\left(i\right)}}{\varphi^{T(i)}\varphi^{\left(i\right)}}\{{℘}^{\left(i\right)}-\varphi^{\left(i\right)}{\hat{\wedge }}^{\left(i-1\right)}-\zeta^{\left(i\right)}\} \end{array}$$

where $\varphi^{T}=[\begin{array}{cccc} e & \;\dot{e} & \;\cdots & \;e^{\left(n-2\right)} \end{array}]$, *ζ* = *e*^(*n*−1)^ and $\hat{\wedge }$ is estimation of ∧. Fig 2 shows structure of Adaptive Feedback Linearization embedded with FRANF. The control law requires estimated $\hat{f}\left(x\right)$ and $\hat{g}\left(x\right)$ of Eq (7) which are identified using NeuroFuzzy identification.

**Fig 3:**
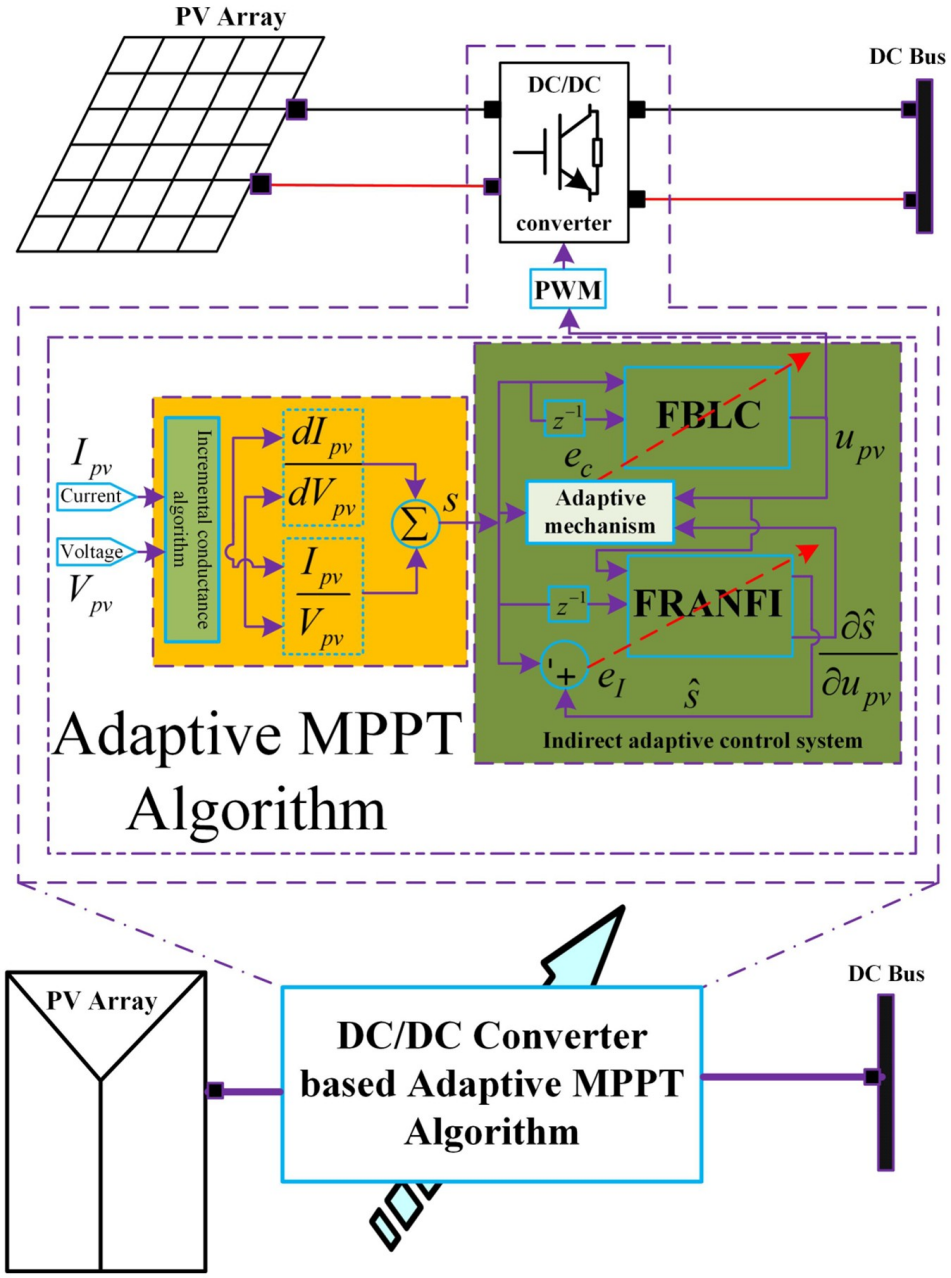
Adaptive FBL embedded FRANF control for MPPT of PV system.

Fig 3 shows the internal control system and the closed-loop adaptive feedback control strategy.

### 2.2 Full recurrent adaptive NeuroFuzzy identification techniques based on different NeuroFuzzy architectures

A variety of FRANF identifiers are used in order to identify the nonlinear $\hat{f}\left(x\right)$ and $\hat{g}\left(x\right)$ functions for PV system in SMG-HPS. The seven-layered FRANF system uses NeuroFuzzy concept for estimation.

Fuzzy logic uses IF-THEN rules for approximation of unknown functions using standard fuzzy model. The unknown functions $\hat{f}\left(x\right)$ and $\hat{g}\left(x\right)$ can be identified by the standard fuzzy model using a set of rules as;

*R*^*m*^: IF *x*_1_ is $A_{1}^{j}\ldots$ and *x*_*n*_ is $A_{n}^{j}$ THEN *y* is $\beta_{l}^{j}$.

Let fuzzy logic controller has *q* inputs, *ρ*_1_, *ρ*_2_, …, *ρ*_*q*_. The output of NeuroFuzzy system is given as:

$$\begin{array}{ccc} ϒ & = & \frac{\sum_{l=1}^{m}\prod_{j=1}^{q}\mu_{F_{j}^{l}}\left(\rho_{j}\right)\beta_{l}}{\sum_{l=1}^{m}\prod_{j=1}^{q}\mu_{F_{j}^{l}}\left(\rho_{j}\right)} \end{array}$$ (10)

where $\mu_{F_{j}^{l}}$ is the membership function, *ρ*_*j*_ and *β*_*l*_ are adjustable parameters. It is the point in *R* at which $\mu_{\beta_{j}}$ achieves its maximum value. *m* is the number of fuzzy rules used to construct the identifier, $F_{j}^{l}$ is the *j*th fuzzy set corresponding to the *l*th fuzzy rule, and *β*_*l*_ is centroid of the *l*th fuzzy set corresponding to identifier output, $\hat{f}\left(x\right)$ and $\hat{g}\left(x\right)$. Eq (10) can be written for $\hat{f}\left(x\right)$ and $\hat{g}\left(x\right)$ using fuzzy-basis function vector *ξ*(*x*), as

$$\begin{array}{ccc} \hat{f}\left(x\right) & = & \beta_{f}^{T}\xi \left(x\right) \end{array}$$ (11)

and

$$\begin{array}{ccc} \hat{g}\left(x\right) & = & \beta_{g}^{T}\xi \left(x\right) \end{array}$$ (12)

where

$$\begin{array}{ccc} \beta_{f} & = & {\left[\beta_{f1}\;\beta_{f2}\;\ldots \;\beta_{fm}\right]}^{T} \end{array}$$ (13)

and

$$\begin{array}{ccc} \beta_{g} & = & {\left[\beta_{g1}\;\beta_{g2}\;\ldots \;\beta_{gm}\right]}^{T} \end{array}$$ (14)

and *ξ*(*x*) is given as

$$\begin{array}{ccc} \xi & = & {\left[\xi_{1}\;\xi_{2}\;\ldots \;\xi_{m}\right]}^{T}\\ & = & \left[\frac{\prod_{j=1}^{q}\mu_{F_{j}^{1}}\left(\rho_{j}\right)}{\sum_{l=1}^{m}\prod_{j=1}^{q}\mu_{F_{j}^{l}}\left(\rho_{j}\right)}\;\ldots \;\frac{\prod_{j=1}^{q}\mu_{F_{j}^{l}}\left(\rho_{j}\right)}{\sum_{l=1}^{m}\prod_{j=1}^{q}\mu_{F_{j}^{l}}\left(\rho_{j}\right)}\right] \end{array}$$ (15)

A number of mathematical relations and functions are available for designing a fast and robust NeuroFuzzy identifier. The following variants are used to design antecedent and the consequent part of the fuzzy logic system for this research work.

#### 2.2.1 Antecedent part

The transformation of continuous input variables into linguistic variables is fuzzification. A membership function is always required for the transformation. The importance of the membership function is based on its shape that translates complete information of the plant (uncertainties and nonlinearities) in fuzzy inference system. The membership function chosen for this research work is the Gaussian membership function due to the following properties:

- local and nonlinear nature
- smooth output
- relation between the radial basis functions Neural Networks (NNs) and fuzzy system

Gradient-based techniques are highly suitable for use due to the continuous differentiable nature of Gaussian membership function. It is expressed as:

$$\begin{array}{cc} \mu_{j}^{r}\left(x_{i}\right) & =exp[-{\left(\frac{x_{i}(k)+m_{ij}}{\sigma_{ij}}\right)}^{2}] \end{array}$$ (16)

where *m*_*ij*_ and *σ*_*ij*_ are the mean and variance of the *i*th input and *j*th membership function.

#### 2.2.2 Variants of consequent part

The consequent part generates weights based on different mathematical functions like Fourier series function, wavelet networks and polynomial NNs. The operation of consequent part takes place in parallel to antecedent part and produces final output of identifier at defuzzification layer. The variants of consequent part used for this research work are Standard Additive Model (SAM), Fourier series function and wavelet networks (Mexican hat wavelet and Chebyshev wavelet).

**Fig 4:**
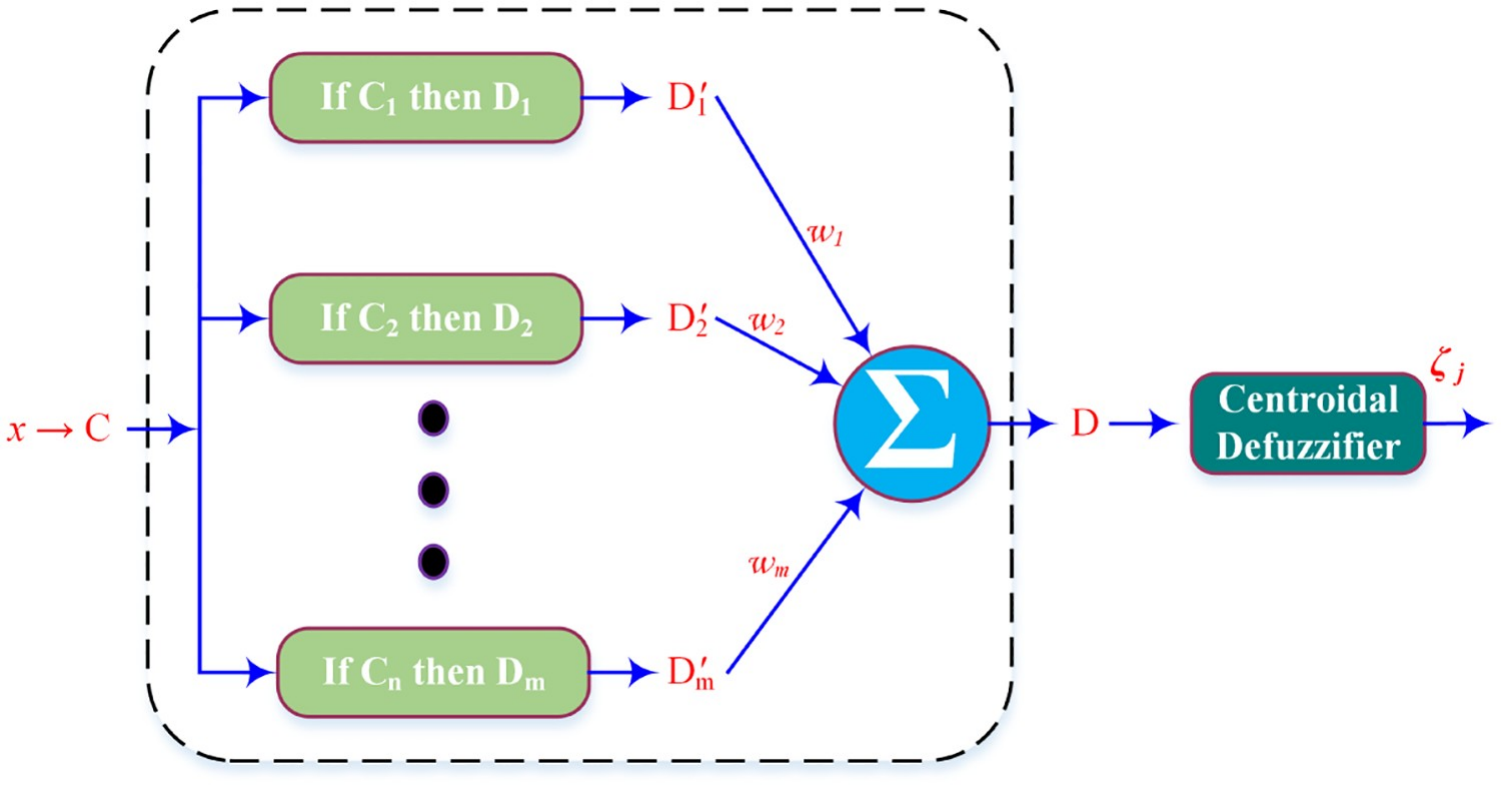
Standard Additive Model architecture.

*2.2.2.1 Standard Additive Model (SAM)*. SAM is an important case of additive fuzzy systems that can estimate any uninterrupted function uniformly over a closed space. In SAM the fuzzy rules are given as:

If *x*_*i*_ = *C* Then *y*_*i*_ = *D* such that *C* and *D* are one to one mapping of input and output spaces. Fig 4 shows the architecture of SAM. Following theorem defines SAM.

**Theorem 1.1** *Consider the fuzzy system* $F:R^{p}\to R^{q}$ *is SAM: F(x) = Centeroid(D) = Centeroid* $\left(\sum_{k=1}^{n}w_{k}a_{k}\left(x\right)D_{k}\right)$ *Then F(x) represents convex sum of n then-part set centroids*;

$$\begin{array}{ccc} F(x) & = & \sum_{k=1}^{n}p_{k}\left(x\right)c_{k} \end{array}$$ (17)

where,

$$\begin{array}{ccc} \;p & = & \frac{w_{k}a_{k}\left(x\right)V_{k}}{\sum_{m=1}^{n}w_{m}a_{m}\left(x\right)V_{m}} \end{array}$$ (18)

*are the convex coefficients or discrete probability weights*, *c*_*k*_ is the centroid of the then-part set and *V*_*k*_ *is the finite positive volume*.

*2.2.2.2 Fourier Series Neural Networks (FsNNs)*. Fast convergence and accurate modeling capabilities of Fourier series NNs are known with gradient descent algorithm. Mutually orthogonal sine and cosine are the basis functions of FsNNs that guarantee better estimation and convergence.

**Fig 5:**
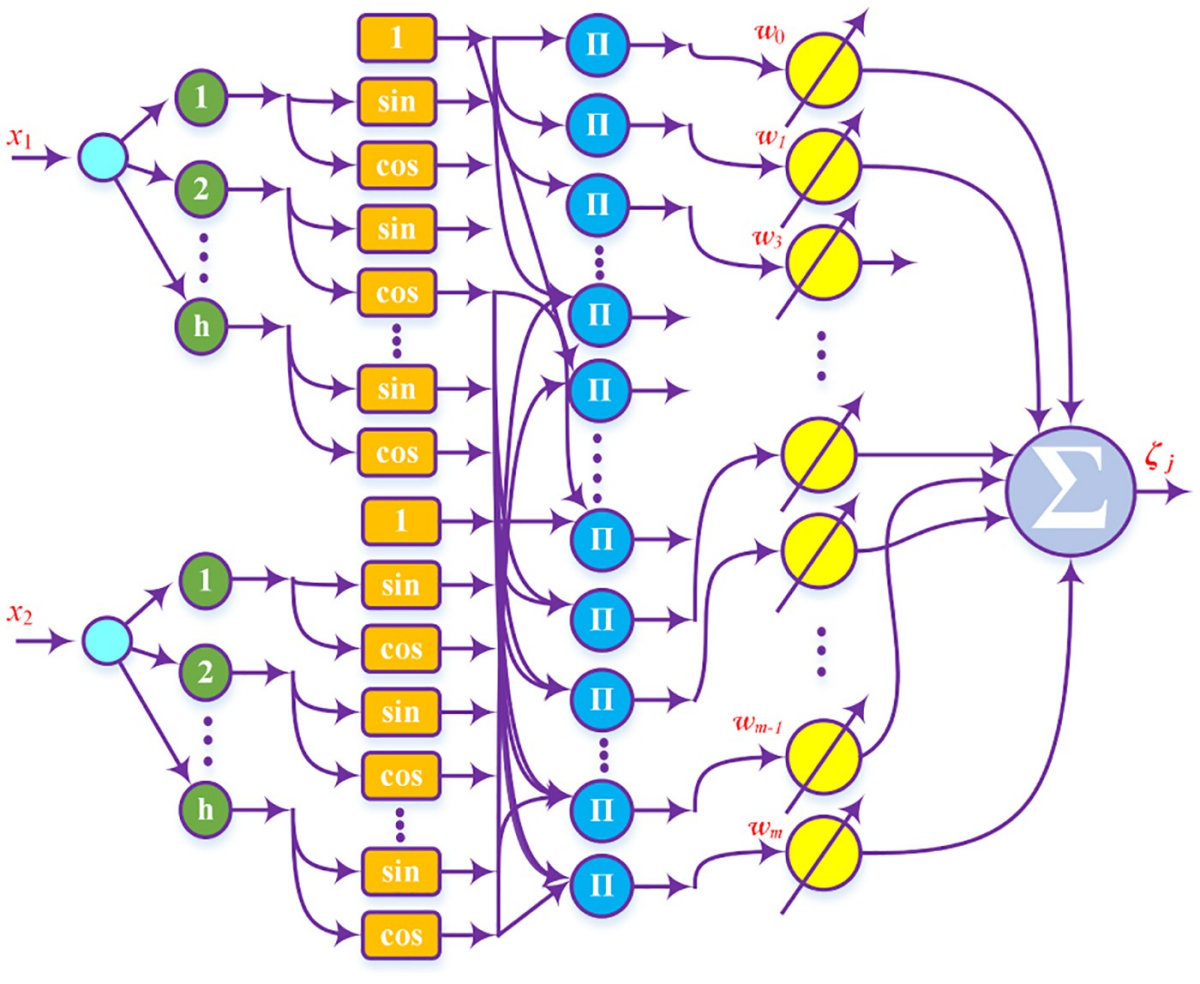
Fourier Series Neural Network.

In FsNN, the *j*th input signal initiates $\frac{p_{j}}{2}-1$ neurons with equal number of sine and cosine as activation function. Adaptation of output weights occur during learning. Overall output of the FsNN is given as:

$$\begin{array}{ccc} f_{i}^{\left(s\right)} & = & \xi (t_{j})\\ & = & [w_{1}\;w_{2}\ldots w_{l}][{\left[1\text{sin}\left(t_{1}\right)\text{cos}\left(t_{1}\right)\text{sin}\left(2t_{1}\right)\text{cos}\left(2t_{1}\right)\ldots \text{sin}\left(pt_{1}\right)\text{cos}\left(pt_{1}\right)\right]}^{T}\\ & & ⊗{\left[1\text{sin}\left(t_{2}\right)\text{cos}\left(t_{2}\right)\text{sin}\left(2t_{2}\right)\text{cos}\left(2t_{2}\right)\ldots \text{sin}\left(pt_{2}\right)\text{cos}\left(pt_{2}\right)\right]}^{T}⊗\cdots ⊗\\ & & {\left[1\text{sin}\left(t_{n}\right)\text{cos}\left(t_{n}\right)\text{sin}\left(2t_{n}\right)\text{cos}\left(2t_{n}\right)\ldots \text{sin}\left(pt_{n}\right)\text{cos}\left(pt_{n}\right)\right]}^{T}] \end{array}$$ (19)

where, ⊗ denotes *Kronecker* product, *n* shows the total number of inputs and $l=\prod_{i=1}^{n}(p_{i}-1)$. Fig 5 shows structure of FsNN. Sine and cosine are the basis functions of FsNNs that span over the infinite interval of time. They are not localized in time and have infinite energy.

**Fig 6:**
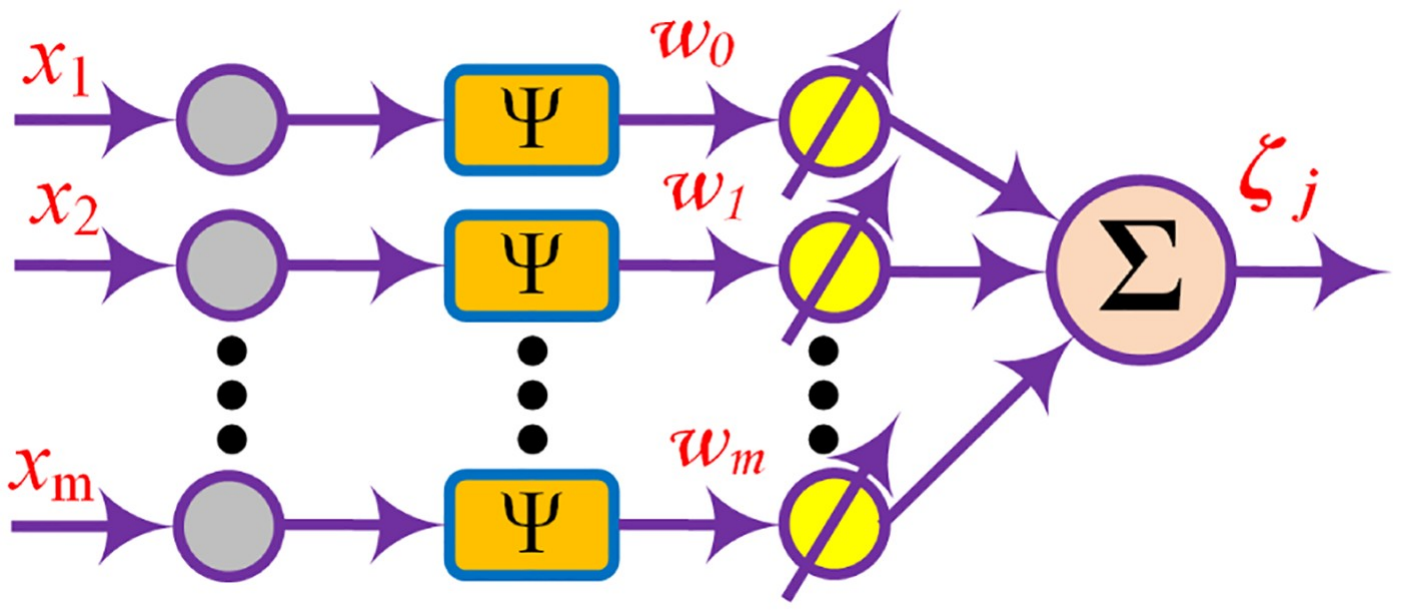
Wavelet Neural Network.

*2.2.2.3 Fuzzy Wavelet Neural Networks (NNs)*. For a better estimation of nonlinear functions, wavelet NNs were proposed as a substitute to feedforward NNs. Due to enormous neurons, NNs may get struck on the local minima that result in slower convergence of the network. To get rid of it, wavelet functions (WFs) can be used in the structure. Wavelets are waves having a limited duration and zero mean value. The localization characteristics of wavelets and the fast learning abilities of NNs results in better outcomes for complex nonlinear system modeling. The schematic diagram of wavelet NN is given in Fig 6. Two wavelet activation functions, Mexican hat and Chebyshev wavelet are used in this research work.

- *Mexican hat wavelet* (MHW) is a negative normalized, non-orthogonal second derivative of Gaussian function. MHW function is expressed as;
  $$\begin{array}{ccc} \Psi_{i}(x_{i}) & = & {\left|d_{ij}\right|}^{-\frac{1}{2}}\psi (\frac{x_{i}-t_{ij}}{d_{ij}})\\ & & d_{ij}\neq 0,\;i=1,2,\ldots n \end{array}$$ (20)
  where Ψ_*i*_(*x*_*i*_) is the family of wavelets obtained by single *ψ*(*x*_*i*_) function with parameters dilation *d*_*ij*_ and *t*_*ij*_ respectively.
- *Chebyshev wavelet* (CW) $\Psi_{rs}\left(t\right)=\psi \left(k,\hat{r},s,t\right)$ have four arguments; *k* *ϵ* N, *r* = 1, 2, …, 2^*k*−1^, and $\hat{r}=2r-1$; *s* is the degree of the first kind Chebyshev polynomial and *t* *ϵ* [0, 1] is the normalized time. They can be expressed on the interval [0, 1] as:
  $$\begin{array}{c} \Psi_{rs}\left(t\right)=\{\begin{array}{c} \begin{array}{c} 2^{k/2}{\tilde{T}}_{s}(2^{k}t-\hat{r}),\\ 0, \end{array}\begin{array}{c} \frac{\hat{r}-1}{2^{k}}\leq t\leq \frac{\hat{r}+1}{2^{k}}\\ otherwise \end{array} \end{array} \end{array}$$ (21)
  where
  $$\begin{array}{c} {\tilde{T}}_{s}\left(t\right)=\{\begin{array}{cc} \begin{array}{c} \frac{1}{\sqrt{\pi }},\\ \sqrt{\frac{2}{\pi }}T_{s}\left(t\right), \end{array} & \begin{array}{c} s=0\\ s>0 \end{array} \end{array} \end{array}$$ (22)
  *s* = 0, 1, …*N* − 1, where *N* is a fixed positive integer. Orthonormality of the system is given by the coefficients in Eq (22). Here nth degree Chebyshev polynomials orthogonal to $w\left(t\right)=1/\sqrt{1-t^{2}}$ weight function on the interval [−1, 1] is represented by $\left\{T_{s}\left(t\right),\;s\epsilon N\cup \left\{0\right\}\right\}$ that satisfy the following recursive formula:
  $$\begin{array}{ccc} T_{o}\left(t\right) & = & 1, \end{array}$$ (23)
  $$\begin{array}{ccc} T_{1}\left(t\right) & = & t,\\ T_{s+1}\left(t\right) & = & 2tT_{s}\left(t\right)-T_{s-1}\left(t\right),\;s=1,2,3\ldots \end{array}$$ (24)
  The weight functions of CW, $\tilde{w}\left(t\right)=w\left(2t-1\right)$ generates orthogonal wavelets on dilation and translation as $w_{s}\left(t\right)=w\left(2^{k}t-\hat{r}\right)$.

#### 2.2.3 Proposed FRANF identifier

**Fig 7:**
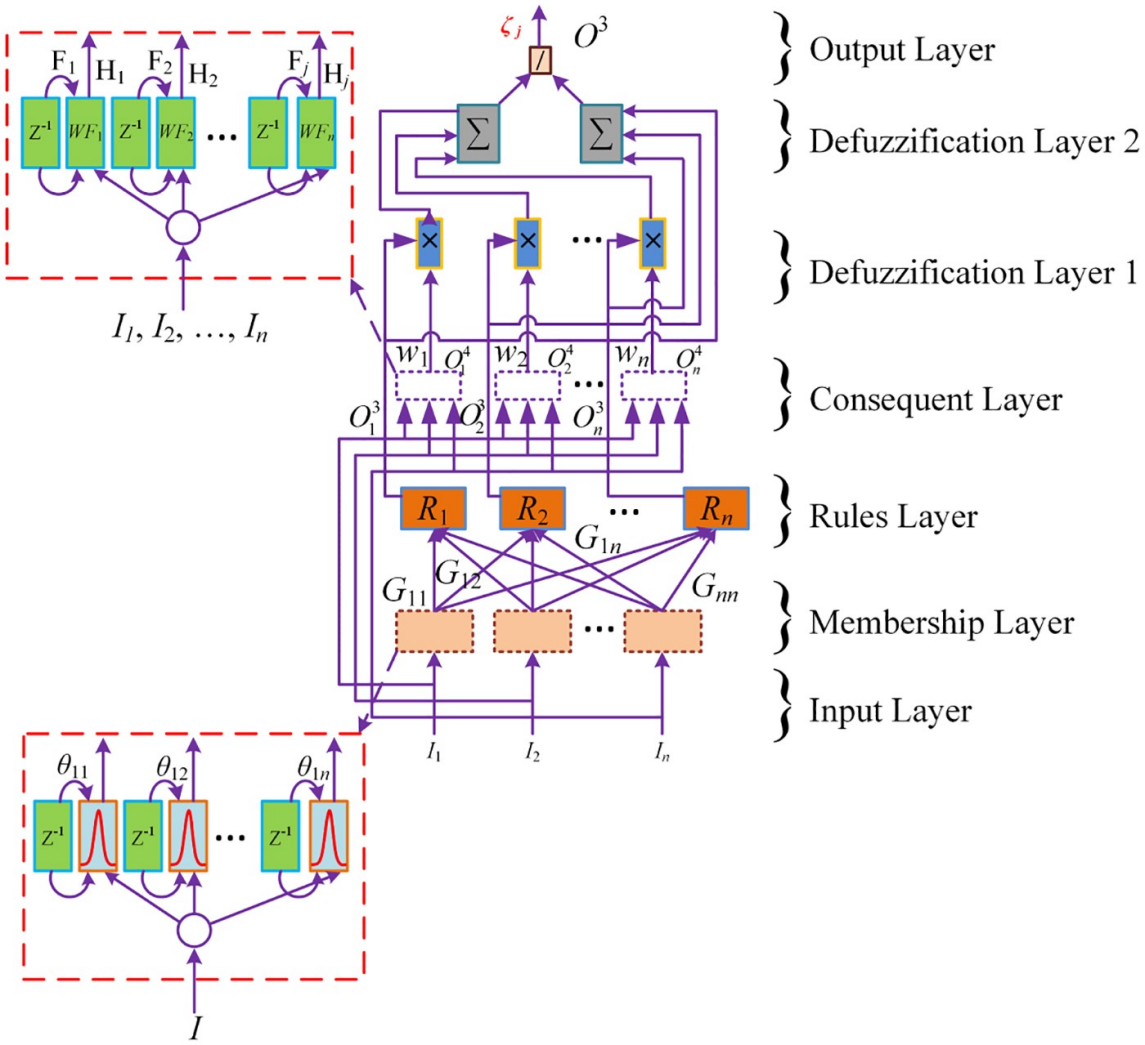
Architecture of Full Recurrent Adaptive NeuroFuzzy System.

The FRANF has seven layers as shown in Fig 7. The antecedent part consists of the first three layers, whereas the rest four layers are consequent part layers. The *n* number of input signals in the first layer is equivalent to the *m* number of nodes and these nodes are used for input distribution. Let $I_{i}^{k}$ and $O_{i}^{k}$ represents input and output of *i*th node in *k*th layer. The operation function of nodes and the signal propagation in each layer is given as under:

- ***Layer 1 (Input Layer)***: It takes input variables and these inputs are transmitted to the next layer by the nodes. Feedback connections are the part of this layer that add temporal relationship in the network.
  $$\begin{array}{ccc} \text{Input}\;\text{is:}\;I_{i}^{1}\left(k\right) & = & x_{i}^{1}\left(k\right) \end{array}$$ (25)
  $$\begin{array}{ccc} \text{Output}\;\text{is:}\;O_{i}^{1}\left(k\right) & = & I_{i}^{1}\left(k\right)=x_{i}^{1}\left(k\right) \end{array}$$ (26)
  where, *i* = 1, 2, 3, ……*n*; *k* is number of iterations.
- ***Layer 2 (Membership Layer)***: Each node represents one linguistic term and computes membership degree and fuzzy set for all input signals entering into the system. Linguistics terms in each node are computed using Gaussian membership function.
  $$\begin{array}{ccc} \text{Input}\;\text{is:}\;I_{i}^{2}\left(k\right) & = & O_{i}^{1}\left(k\right)+O_{i}^{2}\left(k-1\right)\theta_{i}^{2}=x_{i}^{1}\left(k\right)+\mu_{i}^{2}\left(k-1\right)\theta_{i}^{2} \end{array}$$ (27)
  $$\begin{array}{ccc} \text{Output}\;\text{is:}\;O_{i}^{2}\left(k\right) & = & \mu_{i}^{2}=e^{-\frac{1}{2}{\left(\frac{I_{i}^{2}\left(k\right)-m_{i}}{\sigma_{i}}\right)}^{2}} \end{array}$$ (28)
  Where *ij* subscript shows the *j*th term of the *i*th input $O_{i}^{1}$, where j=1,…,N, and the superscript (2) represents second layer. Also, *i* = 1, 2, 3, ……*n*; *k* is number of iterations, $\mu_{i}^{2}(k-1)$ is the past value of membership function and $\theta_{ij}^{2}$ is the recurrent weight.
- ***Layer 3 (Rule Layer)***: The rule layer is just the product of membership functions. The number of rules, i.e. *R*_1_, *R*_2_, …*R*_*n*_ in this layer are equal to number of nodes and each node corresponds to one fuzzy rule. Min operator is used to compute the output signal’s value in each rule. Each $O_{i}^{3}\left(k\right)$ is the input for the next (consequent) layer.
  $$\begin{array}{ccc} \text{Input}\;\text{is:}\;I_{i}^{3}\left(k\right) & = & O_{i}^{2}\left(k\right)=\mu_{i}^{2}=e^{-{\left(\frac{x_{i}^{1}\left(k\right)+\mu_{i}^{2}\left(k-1\right)\theta_{ij}^{2}-m_{i}^{2}}{2\sigma_{i}^{2}}\right)}^{2}} \end{array}$$ (29)
  $$\begin{array}{ccc} \text{Output}\;\text{is:}\;O_{i}^{3}\left(k\right) & = & \mu_{i}^{3}=\overset{n}{\prod_{i=1}}\mu_{i}^{2}=\overset{n}{\prod_{i=1}}e^{-{\left(\frac{x_{i}^{1}\left(k\right)+\mu_{i}^{2}\left(k-1\right)\theta_{ij}^{2}-m_{ij}^{2}}{2\sigma_{ij}^{2}}\right)}^{2}} \end{array}$$ (30)
- ***Layer 4 (Consequent Layer)***: It determines the difference in the proposed control techniques. The general description of this layer is;
  $$\begin{array}{ccc} \text{Input}\;\text{is:}\;I_{i}^{4}\left(k\right) & = & O_{i}^{1}\left(k\right)+O_{i}^{4}\left(k-1\right)F_{ij}^{4}=O_{i}^{1}\left(k\right)+H_{i}F_{ij}^{4} \end{array}$$ (31)
  where *H*_*i*_ is the output of the hidden layer (previous values of mathematical function), and *F*_*i*_ is the feedback weight and the superscript (4) denotes the layer 4. Each mathematical function is multiplied by the weight, *w*_*i*_ of the NNs in fourth layer.
  $$\begin{array}{ccc} \text{Output}\;\text{is:}\;O_{i}^{4}\left(k\right) & = & \beta_{i}^{4}=w_{i}\underset{i=1}{\overset{n}{\Sigma }}\exists_{i}\left(I_{i}^{4}\right) \end{array}$$ (32)
  where $\exists_{i}\left(I_{i}^{4}\right)$ represents one of the used mathematical function discussed in section 2.2.2.
- ***Layer 5 (Defuzzification Layer 1)***: The product of the outputs of antecedent and consequent parts for each input is taken in this layer and then added to each other.
  $$\begin{array}{ccc} \text{Output}\;\text{is:}\;O_{i}^{5}\left(k\right) & = & \underset{i=1}{\overset{n}{\Sigma }}\beta_{i}^{4}\mu_{i}^{3} \end{array}$$ (33)
- ***Layer 6 (Defuzzification Layer 2)***: In this layer the summation of rules (output of antecedent) part is calculated.
  $$\begin{array}{ccc} \text{Output}\;\text{is:}\;O_{i}^{6}\left(k\right) & = & \underset{i=1}{\overset{n}{\Sigma }}\mu_{i}^{3} \end{array}$$ (34)
- ***Layer 7 (Output Layer)***: The final output of the FRANF identifier is estimated in the seventh layer as given below:
  $$\begin{array}{ccc} \text{Output}\;\text{is:}\;O_{i}^{7}\left(k\right) & = & y_{i}=\frac{O_{i}^{5}\left(k\right)}{O_{i}^{6}\left(k\right)}=\frac{\underset{i=1}{\overset{n}{\Sigma }}\beta_{i}^{4}\mu_{i}^{3}}{\underset{i=1}{\overset{n}{\Sigma }}\mu_{i}^{3}} \end{array}$$ (35)

#### 2.2.4 Optimization algorithm

The training of FRANF is for the adjustment of input-output pairs or a given function by fine-tuning network parameters. Mean square error is used as cost function for the training purpose, given as [39–44];

$$\begin{array}{ccc} E & = & \frac{1}{2}\underset{i=1}{\overset{2}{\Sigma }}{\left(y_{i}^{d}-y_{i}\right)}^{2} \end{array}$$ (36)

Gradient descent method is used for fast cost function reduction and convergence [39–44]. The general equation is given as follows:

$$\begin{array}{ccc} \Omega (k+1) & = & \Omega \left(k\right)-\gamma g_{k} \end{array}$$ (37)

where, *g*_*k*_ is the gradient of cost function at *k*th iteration, *γ* > 0 is the learning rate and *k* is the iteration index. The update equations for antecedent part and consequent part are as follows:

*2.2.4.1 Update equations for antecedent part parameters*. The update equation for variants of Gaussian membership function is derived from following chain rule [39–44].

$$\begin{array}{c} \frac{\partial E}{\partial \chi_{i}}=\frac{\partial E}{\partial y_{i}}\frac{\partial y_{i}}{\partial \mu_{i}^{3}}\frac{\partial \mu_{i}^{3}}{\partial \chi_{i}} \end{array}$$ (38)

where, *χ* shows the variant like mean, variance and feedback weight of Gaussian membership function.

Update equation for mean, *m*_*i*_ is;

$$\begin{array}{ccc} m_{i}\left(k+1\right) & = & m_{i}\left(k\right)+\gamma \left(y_{i}^{d}-y_{i}\right)[\frac{\left(\beta_{i}^{4}-\beta_{i+1}^{4}\right)\mu_{i}^{3}\mu_{i+1}^{3}}{{\left(\mu_{i}^{3}+\mu_{i+1}^{3}\right)}^{2}}][\frac{x_{i}^{1}\left(k\right)+\mu_{i}^{2}\left(k-1\right)\theta_{i}^{2}-m_{i}^{2}}{{\left(\sigma_{i}^{2}\right)}^{2}}] \end{array}$$ (39)

Update equation for variance, *σ*_*i*_ is;

$$\begin{array}{ccc} \sigma_{i}\left(k+1\right) & = & \sigma_{i}\left(k\right)+\gamma \left(y_{i}^{d}-y_{i}\right)[\frac{\left(\beta_{i}^{4}-\beta_{i+1}^{4}\right)\mu_{i}^{3}\mu_{i+1}^{3}}{{\left(\mu_{i}^{3}+\mu_{i+1}^{3}\right)}^{2}}][\frac{{\left(x_{i}^{1}\left(k\right)+\mu_{i}^{2}\left(k-1\right)\theta_{i}^{2}-m_{i}^{2}\right)}^{2}}{{\left(\sigma_{i}^{2}\right)}^{3}}] \end{array}$$ (40)

Update equation for recurrent weight, *θ*_*i*_ is;

$$\begin{array}{ccc} \theta_{i}\left(k+1\right) & = & \theta_{i}\left(k\right)-\gamma \left(y_{i}^{d}-y_{i}\right)[\frac{\left(\beta_{i}^{4}-\beta_{i+1}^{4}\right)\mu_{i}^{3}\mu_{i+1}^{3}}{{\left(\mu_{i}^{3}+\mu_{i+1}^{3}\right)}^{2}}][\frac{x_{i}^{1}\left(k\right)+\mu_{i}^{2}\left(k-1\right)\theta_{i}^{2}-m_{i}^{2}}{{\left(\sigma_{i}^{2}\right)}^{2}}] \end{array}$$ (41)

*2.2.4.2 Update equations for consequent part*. Chain rule for updating variants of consequent part is [39–44];

$$\begin{array}{ccc} \frac{\partial E}{\partial \varpi_{i}} & = & \frac{\partial E}{\partial y_{i}}\frac{\partial y_{i}}{\partial \beta_{i}^{4}}\frac{\partial \beta_{i}^{4}}{\partial \exists_{i}}\frac{\partial \exists_{i}}{\partial \varpi_{i}} \end{array}$$ (42)

where ∃_*i*_ represents one of the mathematical functions discussed in section 2.2.2 and ϖ shows the variants of mathematical function like centroid, dilation, translation and volume etc.

Update equation for centroid, *c*_*i*_ of SAM is;

$$\begin{array}{ccc} c_{i}\left(k+1\right) & = & c_{i}\left(k\right)-\gamma \left(y_{i}^{d}-y_{i}\right)\left(w_{i}\right)(\frac{\mu_{i}^{3}}{\mu_{i}^{3}+\mu_{i+1}^{3}})(\frac{w_{i}\left(k\right)a_{i}\left(x_{i}^{1}\right)V_{i}}{\sum_{l=1}^{m}w_{l}a_{l}\left(x_{i}^{1}\right)V_{l}}) \end{array}$$ (43)

where, *w*_*i*_ is the adaptive weight of consequent part obtained by Eq (51), *w*_*i*_(*k*) and *w*_*l*_(*k*) are the weights of SAM. Update equation for volume, *V*_*i*_ of SAM is;

$$\begin{array}{ccc} V_{i}\left(k+1\right) & = & V_{i}\left(k\right)-\gamma \left(y_{i}^{d}-y_{i}\right)\left(w_{i}\right)(\frac{\mu_{i}^{3}}{\mu_{i}^{3}+\mu_{i+1}^{3}})(\frac{w_{i}\left(k\right)a_{i}\left(x_{i}^{1}\right)c_{i}}{\sum_{l=1}^{m}w_{l}\left(k\right)a_{l}\left(x_{i}^{1}\right)V_{l}}) \end{array}$$ (44)

Update equation for weight, *w*_*i*_(*k*) of FsNNs is;

$$\begin{array}{ccc} w_{i}\left(k+1\right) & = & w_{i}\left(k\right)-\gamma \left(y_{i}^{d}-y_{i}\right)\left(w_{i}\right)(\frac{\mu_{i}^{3}}{\mu_{i}^{3}+\mu_{i+1}^{3}})w_{i}\left(k\right) \end{array}$$ (45)

where, *w*_*i*_ is the weight of consequent part from Eq (51), *w*_*i*_(*k*) is the weight of FsNNs.

Chain rule for updating variants of MHW used in this research work is:

$$\begin{array}{c} \frac{\partial E}{\partial \varpi_{i}}=\frac{\partial E}{\partial y_{i}}\frac{\partial y_{i}}{\partial \beta_{i}^{4}}\frac{\partial \beta_{i}^{4}}{\partial \Psi_{i}}\frac{\partial \Psi_{i}}{\partial Z_{i}}\frac{\partial Z_{i}}{\partial \varpi_{i}} \end{array}$$ (46)

where here Ψ_*i*_ represents MHW, *Z*_*i*_ defined below is an intermediate state variable and ϖ shows the variants of MHW like, translation, dilation, feedback weight etc.

$$\begin{array}{c} Z_{i}=(\frac{x_{i}^{1}\left(k\right)+H_{i}F_{i}^{4}-t_{i}}{d_{i}}) \end{array}$$ (47)

Update equation for translation, *t*_*i*_ is;

$$\begin{array}{ccc} t_{i}\left(k+1\right) & = & t_{i}\left(k\right)-\gamma \left(y_{i}^{d}-y_{i}\right)(\frac{\mu_{i}^{3}w_{i}}{\mu_{i}^{3}+\mu_{i+1}^{3}})[e^{-0.5{\left(Z_{i}\right)}^{2}}\frac{\left[\frac{0.5}{Z_{i}}-3.5Z_{i}+{\left(Z_{i}\right)}^{3}\right]}{|d_{i}{|}^{3/2}}] \end{array}$$ (48)

Update equation for dilation, *d*_*i*_ is;

$$\begin{array}{ccc} d_{i}\left(k+1\right) & = & d_{i}\left(k\right)-\gamma \left(y_{i}^{d}-y_{i}\right)(\frac{\mu_{i}^{3}w_{i}}{\mu_{i}^{3}+\mu_{i+1}^{3}})[e^{-0.5{\left(Z_{i}\right)}^{2}}\frac{\left[0.5-3.5Z_{i}^{2}+{\left(Z_{i}\right)}^{4}\right]}{|d_{i}{|}^{3/2}}] \end{array}$$ (49)

Update equation for feedback weight, *F*_*i*_ of MHW is;

$$\begin{array}{ccc} F_{i}\left(k+1\right) & = & F_{i}\left(k\right)+\gamma \left(y_{i}^{d}-y_{i}\right)(\frac{\mu_{i}^{3}w_{i}}{\mu_{i}^{3}+\mu_{i+1}^{3}})[e^{-0.5{\left(Z_{i}\right)}^{2}}\frac{\left[\frac{0.5}{Z_{i}}-3.5Z_{i}+{\left(Z_{i}\right)}^{3}\right]}{|d_{i}{|}^{3/2}}]H_{i} \end{array}$$ (50)

The update equations of CW are applied according to the equations given in section 2.2.2.3.

Weight of consequent part are being updated according to the following chain rule:

$$\begin{array}{c} \frac{\partial E}{\partial w_{i}}=\frac{\partial E}{\partial y_{i}}\frac{\partial y_{i}}{\partial \beta_{i}^{4}}\frac{\partial \beta_{i}^{4}}{\partial w_{i}} \end{array}$$ (51)

where *w*_*i*_ represents the weight of the consequent layer.

$$\begin{array}{ccc} w_{i}\left(k+1\right) & = & w_{i}\left(k\right)+\gamma \left(y_{i}^{d}-y_{i}\right)(\frac{\mu_{i}^{3}}{\mu_{i}^{3}+\mu_{i+1}^{3}})[\exists_{i+1}+\exists_{i}] \end{array}$$ (52)

The feedback weight of antecedent part of all proposed controllers is adaptive while feedback weight of consequent part of only Mexican hat wavelet is adaptive. The feedback weight of rest of the consequent parts of other controllers is closed-loop fixed gain.

### 2.3 Proposed controller Schemes

#### 2.3.1 Adaptive Feedback Linearization (FBL) embedded with Full Recurrent Adaptive NeuroFuzzy (FRANF) Standard Additive Model (SAM) control

This controller is based on the FBL scheme which is embedded with a FRANF-SAM identifier to identify unknown nonlinear functions for FBL. The antecedent part of FRANF is modeled using Gaussian membership function while the consequent part is modeled using SAM as discussed in section 2.2.2.1 and section 2.2.3. The overall explicit control law for this control scheme is given in Eq (53).

uMPPT=[-wfi∑i=1n∑k=1nwfkafk(x)Vfk∑m=1nwfmafm(x)Vfmcfk∏i=1ne-(xfi(k)+μfi(k-1)θfij-mfij22σfij)2-Kv[∧T1]e-YD]wgi∑i=1n∑k=1nwgkagk(x)Vgk∑m=1nwgmagm(x)Vgmcgk∏i=1ne-(xgi(k)+μgi(k-1)θgij-mgij22σgij)2 (53)

#### 2.3.2 Adaptive Feedback Linearization (FBL) embedded with Full Recurrent Adaptive NeuroFuzzy (FRANF) Fourier Series (FS) control

This controller is based on the FBL scheme which is embedded with a FRANF-FS identifier to estimate unknown nonlinear functions for FBL. The antecedent part of FRANF is modeled using Gaussian membership function while the consequent part is modeled using FS as discussed in section 2.2.2.2 and section 2.2.3. The overall explicit control law for this control scheme is given in Eq (54).

$$u_{MPPT}=\frac{[-w_{fi}[w_{f1}\;w_{f2}\ldots w_{fl}][[1\text{sin}\left(t_{f1}\right)\text{cos}\left(t_{f1}\right)\text{sin}\left(2t_{f1}\right)\text{cos}\left(2t_{f1}\right)\ldots \text{sin}\left(pt_{f1}\right)\text{cos}\left(pt_{f1}\right){]}^{T}⊗{\left[1\text{sin}\left(t_{f2}\right)\text{cos}\left(t_{f2}\right)\text{sin}\left(2t_{f2}\right)\text{cos}\left(2t_{f2}\right)\ldots \text{sin}\left(pt_{f2}\right)\text{cos}\left(pt_{f2}\right)\right]}^{T}⊗\ldots ⊗{\left[1\text{sin}\left(t_{fn}\right)\text{cos}\left(t_{fn}\right)\text{sin}\left(2t_{fn}\right)\text{cos}\left(2t_{fn}\right)\ldots \text{sin}\left(pt_{fn}\right)\text{cos}\left(pt_{fn}\right)\right]}^{T}]\prod_{i=1}^{n}e^{-{\left(\frac{x_{fi}\left(k\right)+\mu_{fi}\left(k-1\right)\theta_{fij}-m_{fij}^{2}}{2\sigma_{fij}}\right)}^{2}}-K_{v}[\wedge^{T}\;\;\;1]e-Y_{D}]}{[w_{gi}[w_{g1}\;w_{g2}\ldots w_{gl}][[1\text{sin}\left(t_{g1}\right)\text{cos}\left(t_{g1}\right)\text{sin}\left(2t_{g1}\right)\text{cos}\left(2t_{g1}\right)\ldots \text{sin}\left(pt_{g1}\right)\text{cos}\left(pt_{g1}\right){]}^{T}⊗{\left[1\text{sin}\left(t_{g2}\right)\text{cos}\left(t_{g2}\right)\text{sin}\left(2t_{g2}\right)\text{cos}\left(2t_{g2}\right)\ldots \text{sin}\left(pt_{g2}\right)\text{cos}\left(pt_{g2}\right)\right]}^{T}⊗\ldots ⊗{\left[1\text{sin}\left(t_{gn}\right)\text{cos}\left(t_{gn}\right)\text{sin}\left(2t_{gn}\right)\text{cos}\left(2t_{gn}\right)\ldots \text{sin}\left(pt_{gn}\right)\text{cos}\left(pt_{gn}\right)\right]}^{T}]\prod_{i=1}^{n}e^{-{\left(\frac{x_{gi}\left(k\right)+\mu_{gi}\left(k-1\right)\theta_{gij}-m_{gij}^{2}}{2\sigma_{gij}}\right)}^{2}}]}$$ (54)

#### 2.3.3 Adaptive Feedback Linearization (FBL) embedded with Full Recurrent Adaptive NeuroFuzzy(FRANF) Mexican Hat Wavelet (MHW) control

This controller is based on the FBL scheme which is embedded with a FRANF-MHW identifier to identify unknown nonlinear functions for FBL. The antecedent part of FRANF is modeled using Gaussian membership function while the consequent part is modeled using MHW as discussed in section 2.2.2.3 and section 2.2.3. The overall explicit control law for this control scheme is given in Eq (55).

$$\begin{array}{c} u_{MPPT}=\frac{[-w_{fi}{\left|d_{fij}\right|}^{-\frac{1}{2}}\psi (\frac{x_{fi}-t_{fij}}{d_{fij}})\prod_{i=1}^{n}e^{-{\left(\frac{x_{fi}\left(k\right)+\mu_{fi}\left(k-1\right)\theta_{fij}-m_{fij}^{2}}{2\sigma_{fij}}\right)}^{2}}-K_{v}[\wedge^{T}\;\;\;1]e-Y_{D}]}{{\left|d_{gij}\right|}^{-\frac{1}{2}}\psi (\frac{x_{gi}-t_{gij}}{d_{gij}})\prod_{i=1}^{n}e^{-{\left(\frac{x_{gi}\left(k\right)+\mu_{gi}\left(k-1\right)\theta_{gij}-m_{gij}^{2}}{2\sigma_{gij}}\right)}^{2}}} \end{array}$$ (55)

#### 2.3.4 Adaptive Feedback Linearization (FBL) embedded with Full Recurrent Adaptive NeuroFuzzy (FRANF) Chebyshev Wavelet (CW) control

This controller is based on the FBL scheme which is embedded with a FRANF-CW identifier to identify unknown nonlinear functions for FBL. The antecedent part of FRANF is modeled using Gaussian membership function while the consequent part is modeled using CW as discussed in section 2.2.2.3 and section 2.2.3. The overall explicit control law for this control scheme is given in Eq (56).

$$\begin{array}{c} u_{MPPT}=\begin{array}{c} \frac{[-w_{fi}2^{k/2}{\tilde{T}}_{fs}(2^{k}t-\hat{r})\prod_{i=1}^{n}e^{-{\left(\frac{x_{fi}\left(k\right)+\mu_{fi}\left(k-1\right)\theta_{fij}-m_{fij}^{2}}{2\sigma_{fij}}\right)}^{2}}-K_{v}[\wedge^{T}\;\;\;1]e-Y_{D}]}{{\tilde{T}}_{gs}(2^{k}t-\hat{r})\prod_{i=1}^{n}e^{-{\left(\frac{x_{gi}\left(k\right)+\mu_{gi}\left(k-1\right)\theta_{gij}-m_{gij}^{2}}{2\sigma_{gij}}\right)}^{2}}}, \end{array}\begin{array}{c} \frac{\hat{r}-1}{2^{k}}\leq t\leq \frac{\hat{r}+1}{2^{k}} \end{array} \end{array}$$ (56)

## Results and discussion

The SMG system is developed in Matlab/Simulink R2015a for evaluation of the performance of the proposed controllers. The system is developed by using power generation sources like UG, PV, WT, electrolyzer, SOFC, MT, and the backup sources i.e. SC and batteries whose ratings are given in Table 1. The purpose of using multiple sources is to entertain the dynamic residential load and CS load.

Intelligent supervisory control is an essential part of the SMG system. It monitors the power generation and load variations during the simulation time. The supervisory control ensures the power consumption from renewable resources at a priority level during their availability period. It satisfies the load demand by shifting the load to other sources and UG during peak hours and in the absence of renewable power.

Intelligent control schemes like Adaptive FBL embedded FRANF-SAM, Adaptive FBL embedded FRANF-FS, Adaptive FBL embedded FRANF-MHW, Adaptive FBL embedded FRANF-CW are used to extract the maximum power from PV system connected in the microgrid. The performance of all the proposed control schemes is compared to aPID based MPPT control scheme.

### 3.1 Case studies

Three different cases are taken in this research work, e.g., (a) Step change in both solar irradiation and temperature; (b) Partial shading condition; and (c) Daily field data of solar irradiation and temperature in Islamabad, to evaluate the performance of proposed adaptive FBL embedded FRANF controllers under PSC compared to aPID control scheme.

#### 3.1.1 Step change in both solar irradiation and temperature

**Fig 8:**
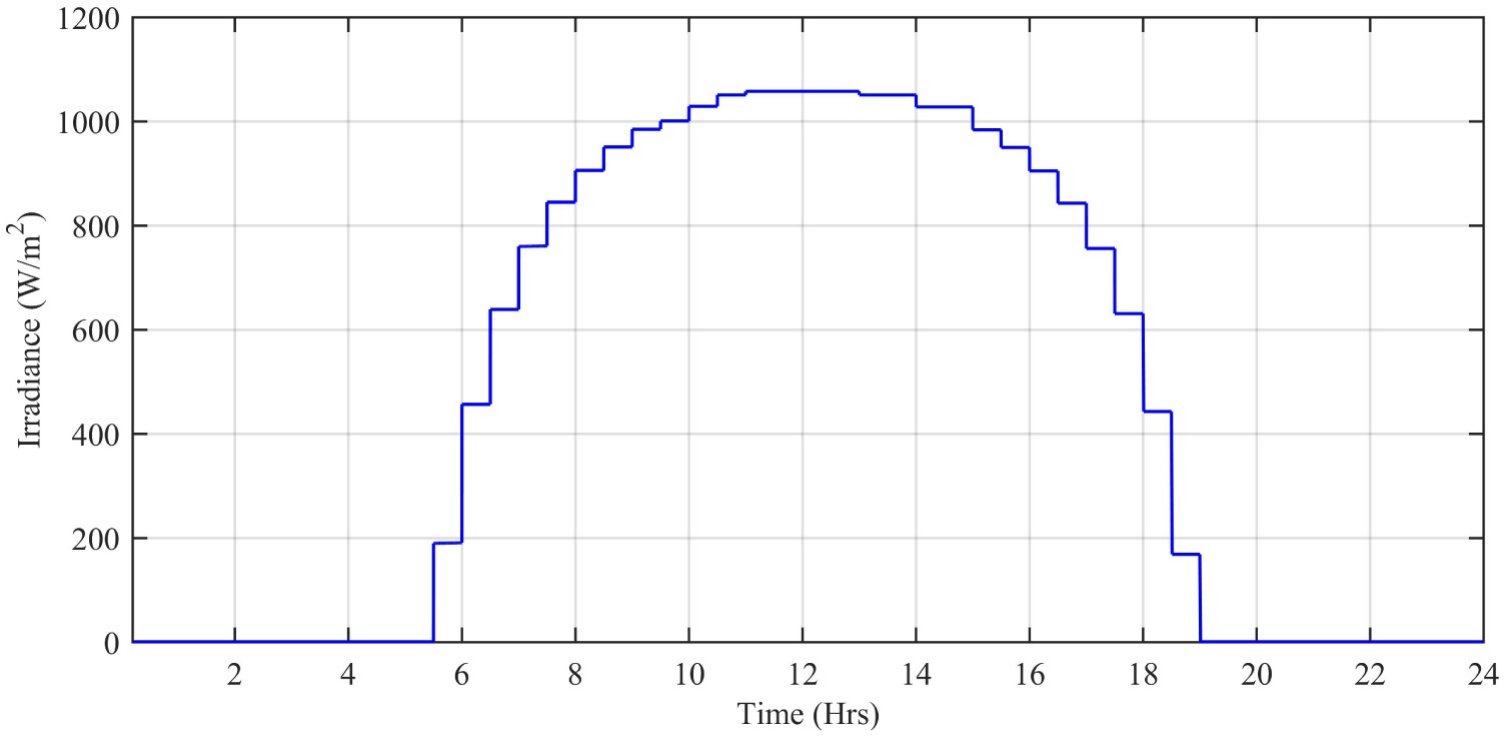
Step profile of solar irradiance level (W/*m*^2^).

**Fig 9:**
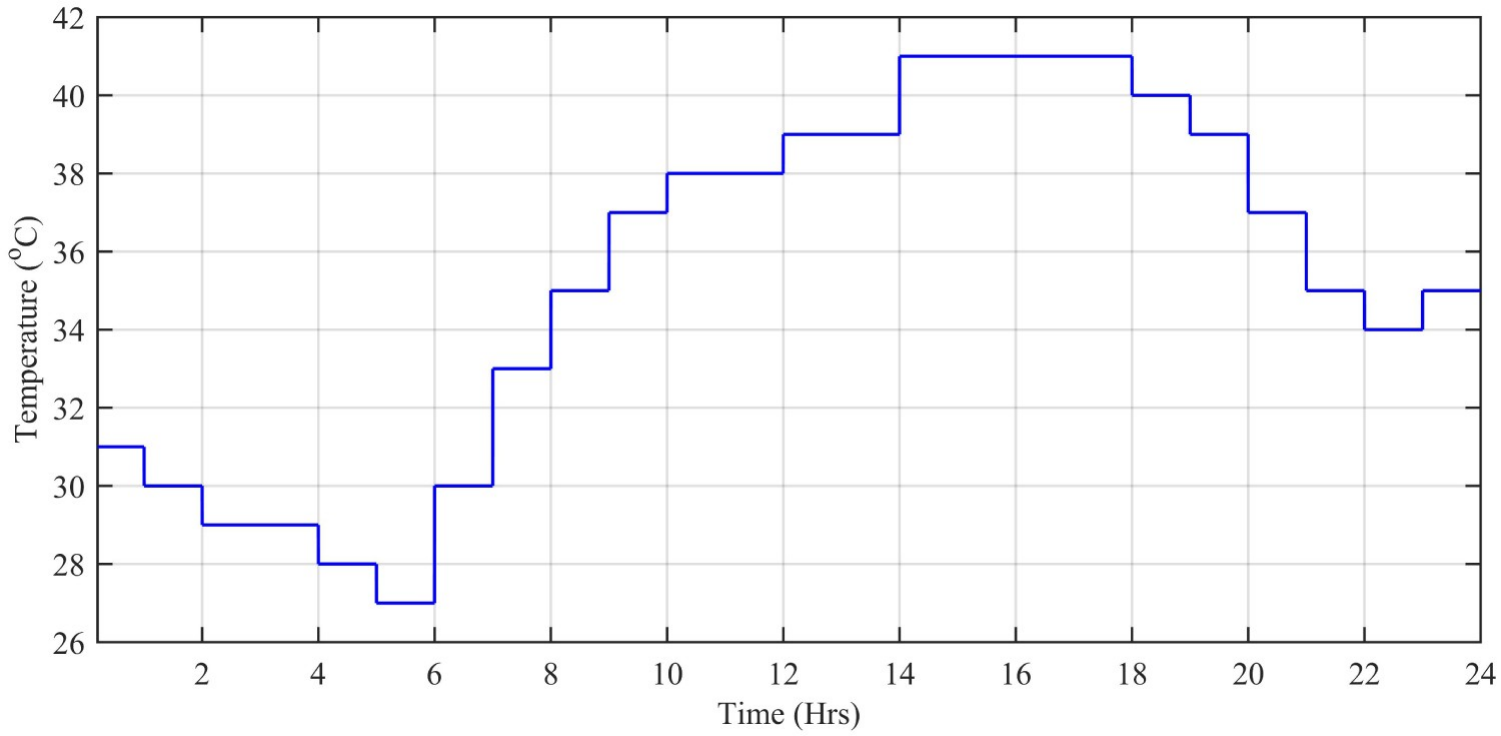
Step profile of ambient temperature level (°C).

Step-changing solar irradiation and temperature profile are simulated for 24 seconds, where each second represents one hour. The solar irradiation is gradually increased in many steps to its maximum and then gradually decreased to zero. Figs 8 and 9 shows the step profile of solar irradiation and ambient temperature used for this case study.

**Fig 10:**
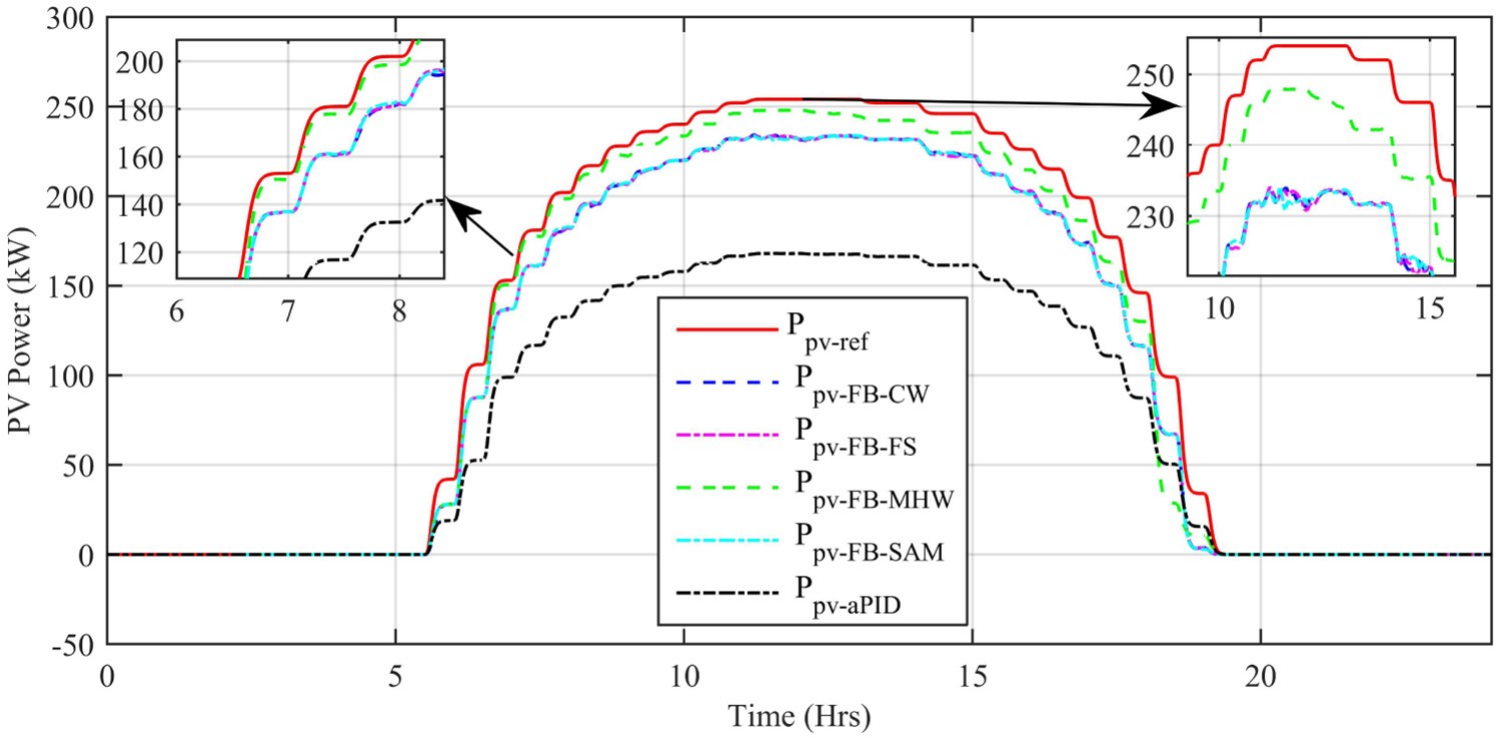
PV MPP tracked vs reference power for step change profile.

Fig 10 shows the output power comparison of all intelligent proposed control schemes with aPID. Every step change is accurately determined by the proposed control schemes and the each proposed control scheme generates more output power compared to aPID. Furthermore, it is also clear that the power generated by three proposed controllers, i.e., adaptive FBL embedded FRANF-CW, adaptive FBL embedded FRANF-FS, and adaptive FBL embedded FRANF- SAM, seems to be very near to each other. However, the power generated by adaptive FBL embedded FRANF-MHW is maximum than other proposed schemes.

Power error (*P*_*error*_) can be described as:

$$\begin{array}{c} P_{error}=P_{ref}-P_{PV} \end{array}$$ (57)

where *P*_*ref*_ is the reference power and *P*_*PV*_ is tracked output power obtained from the PV system under the action of applied controllers. The maximum and average power error of all the control schemes is given in Table 2.

**Table 2:** Peak power error and average power error of all control schemes for step changing solar profile.

Adaptive FBL based Control Scheme	Maximum Peak Power Error (*kW*)	Average Power Error (*kW*)
aPID	38.77	19.46
FRANF-CW	32.17	12.53
FRANF-SAM	32.1	12.51
FRANF-FS	31.96	12.52
FRANF-MHW	70.46	6.514

**Fig 11:**
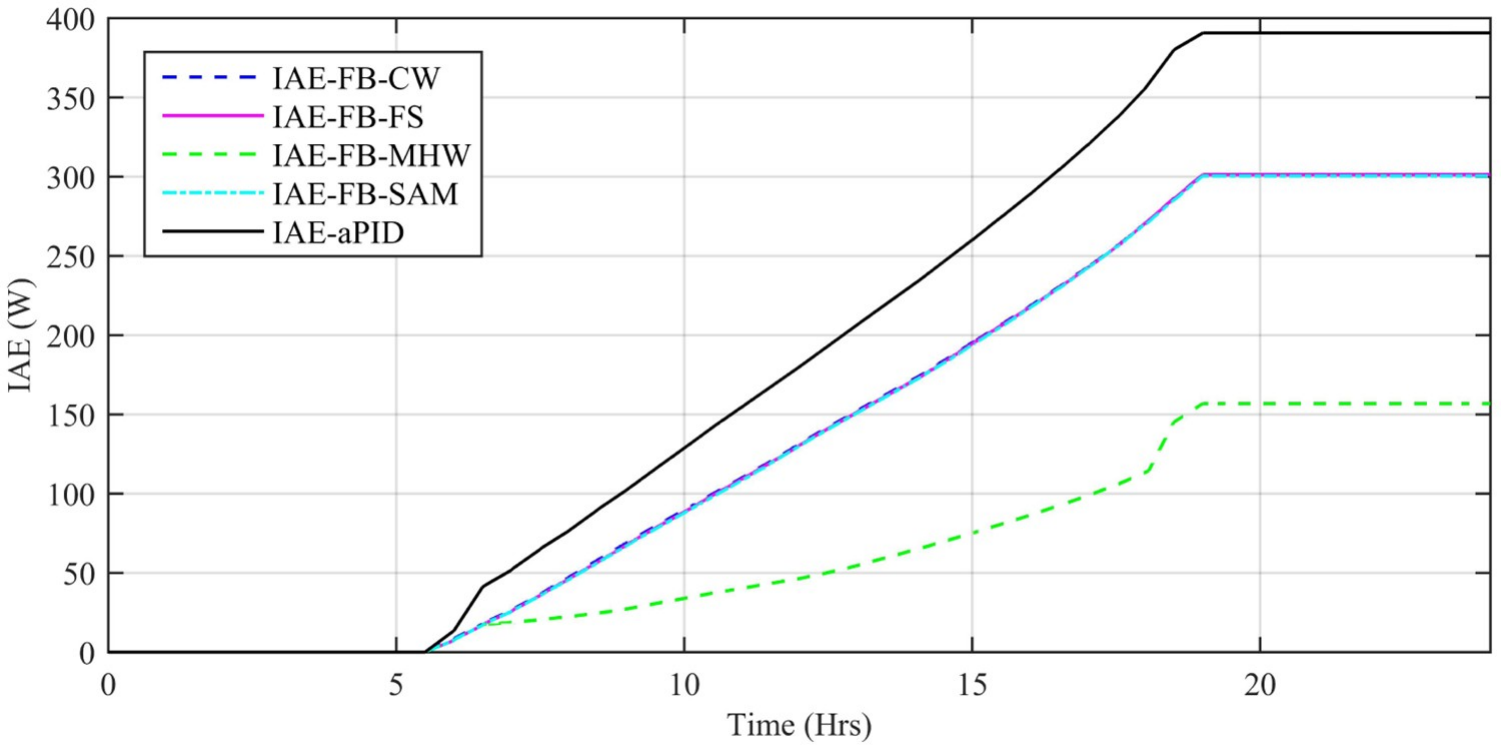
IAE for step changing solar profile.

**Fig 12:**
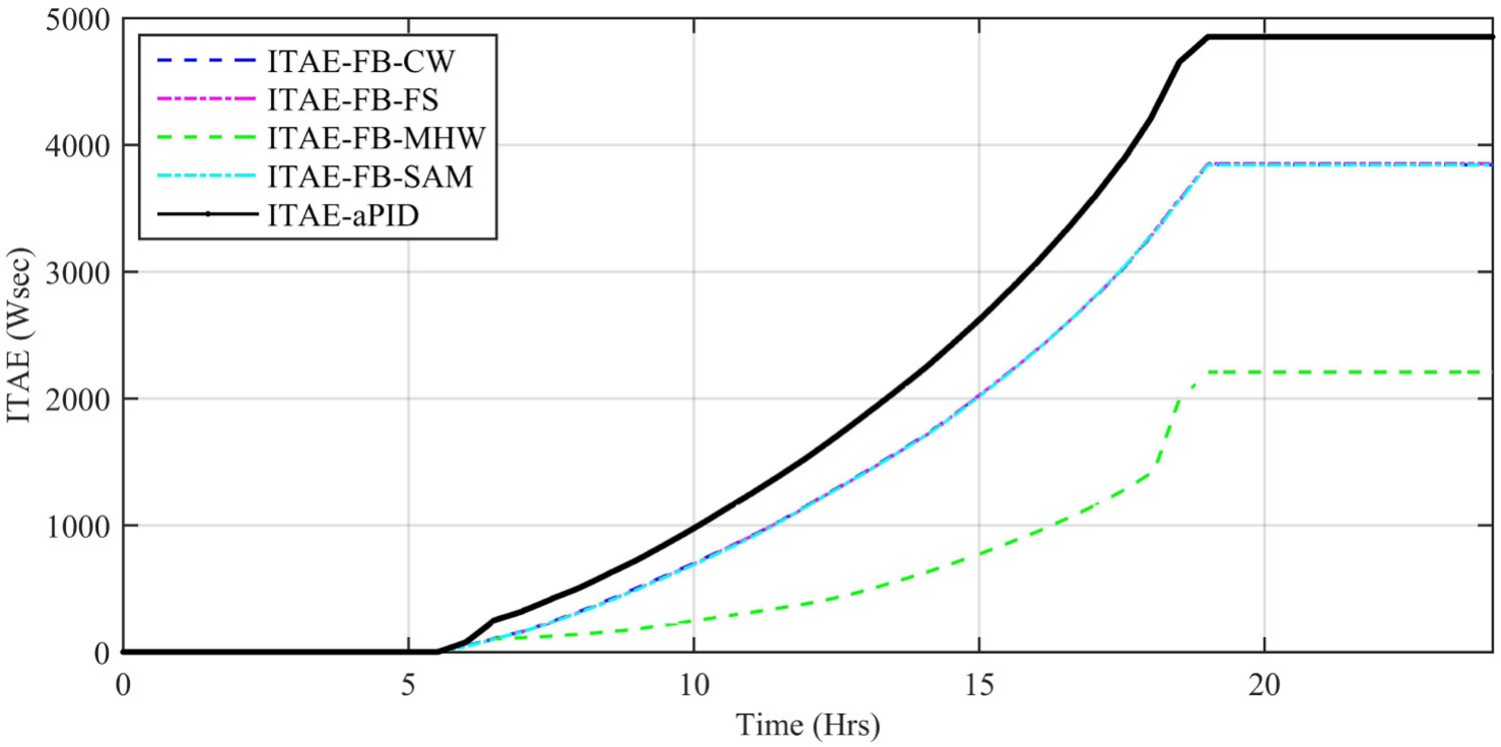
ITAE for step changing solar profile.

**Fig 13:**
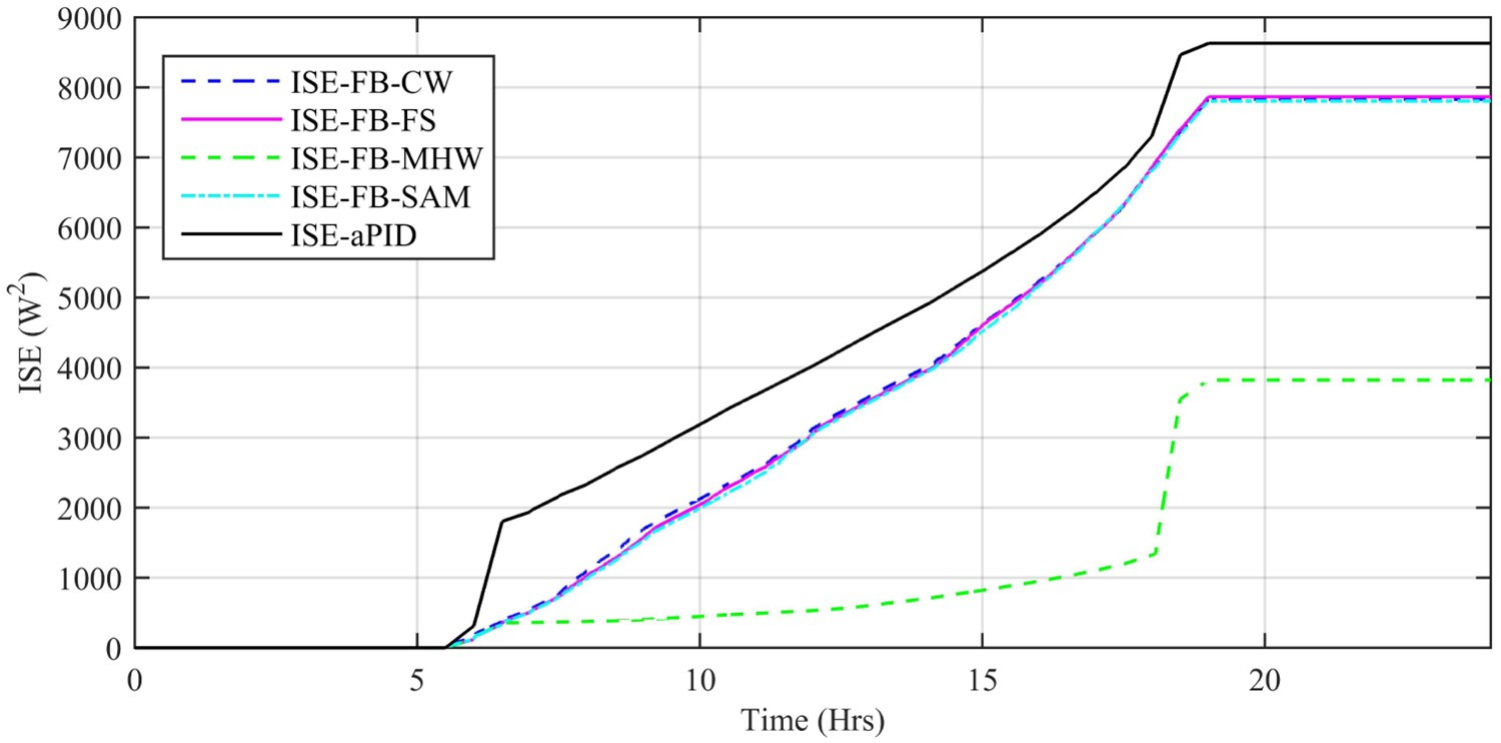
ISE for step changing solar profile.

**Fig 14:**
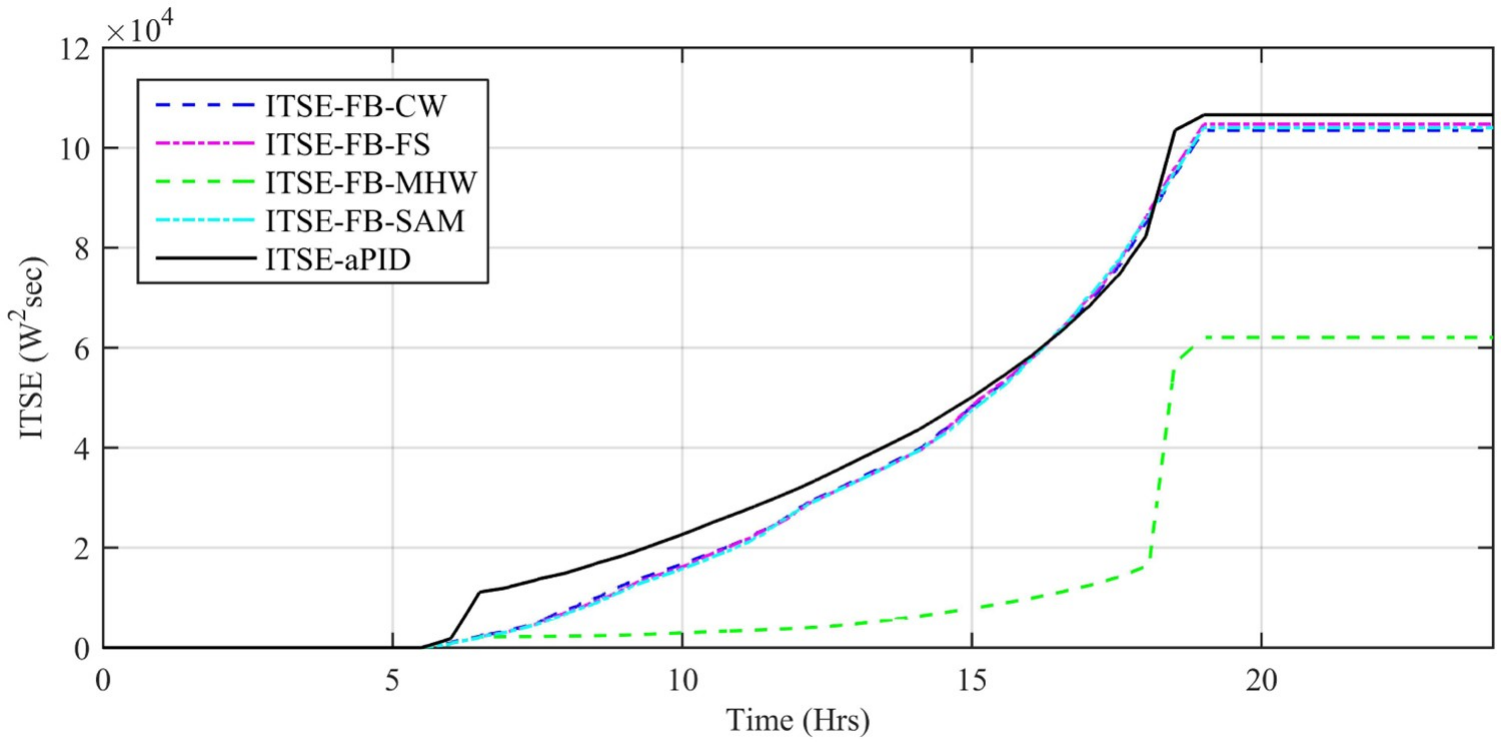
ITSE for step changing solar profile.

Performance indexes including Integral Square Error (ISE), Integral Time Square Error (ITSE), Integral Absolute Error (IAE), and Integral Time Absolute Error (ITAE), calculated based on *P*_*error*_ in Eq (57) as shown in Figs 11, 12, 13 and 14. The comparison of performance indexes plots shows that the accumulative error in all schemes increases with time. The index of adaptive FBL embedded FRANF-MHW is least among all proposed controllers. Table 3 shows the values of various indexes of all the proposed controllers compared to aPID showing least to most performing from top to bottom.

**Table 3:** Performance indexes for step changing solar.

Adaptive FBL based Control Scheme	IAE (W)	ITAE (Wsec)	ISE (W^2^)	ITSE (W^2^sec)
aPID	390.7	4854	8631	106600
FRANF-CW	301.3	3844	7830	103500
FRANF-FS	301.2	3851	7867	104700
FRANF-SAM	300.4	3842	7805	104000
FRANF-MHW	156.8	2208	3823	62050

The switching in the converter circuits arises harmonics in the load voltage and current that are not synchronized with the frequency of the system. It wastes power as heat and should be minimized. Voltage fluctuations and flickers are caused by higher frequency harmonics.

**Fig 15:**
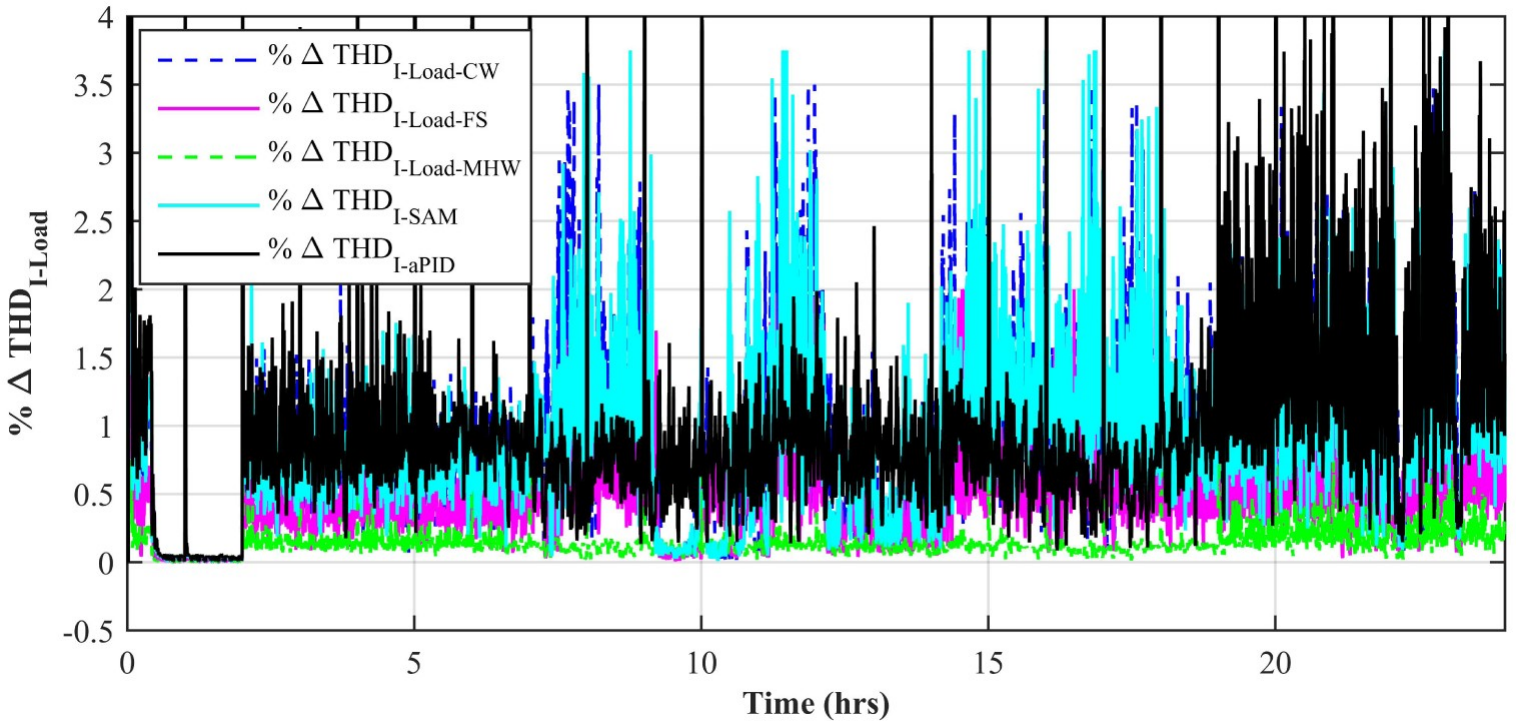
%age THD change in load current for step changing solar profile.

**Fig 16:**
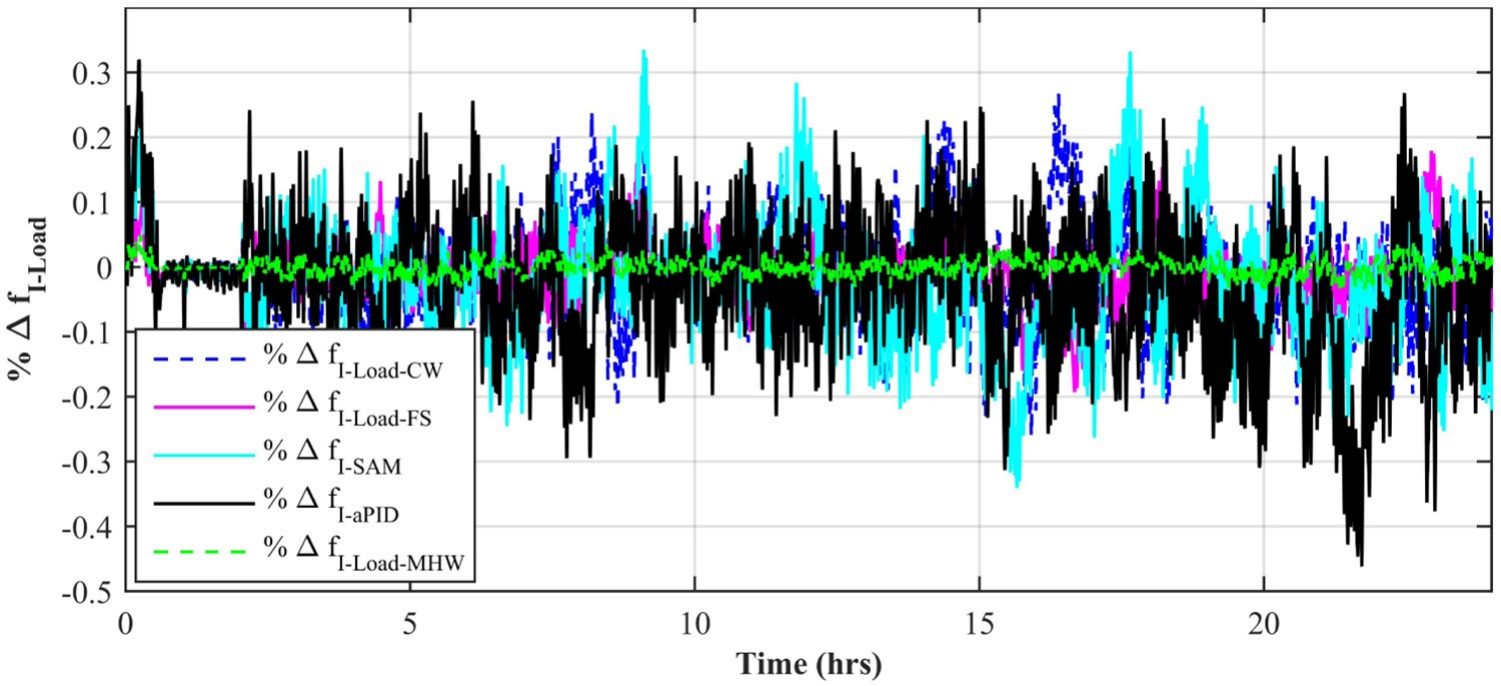
%age frequency change for step changing solar profile.

Fig 15 shows a comparison of the percentage change in total harmonic distortion (THD) for load current due to individual control scheme. The result shows that the percentage change in THD due to the Adaptive FBL embedded FRANF-MHW control scheme is the smallest of all and proves its better performance among other proposed controllers. The percentage change in frequency is also shown in Fig 16 for all proposed controllers. It can be observed that the percentage change in frequency due to the adaptive FBL embedded FRANF-MHW control scheme is almost flat and nearly zero.

#### 3.1.2 Partial shading condition

**Fig 17:**
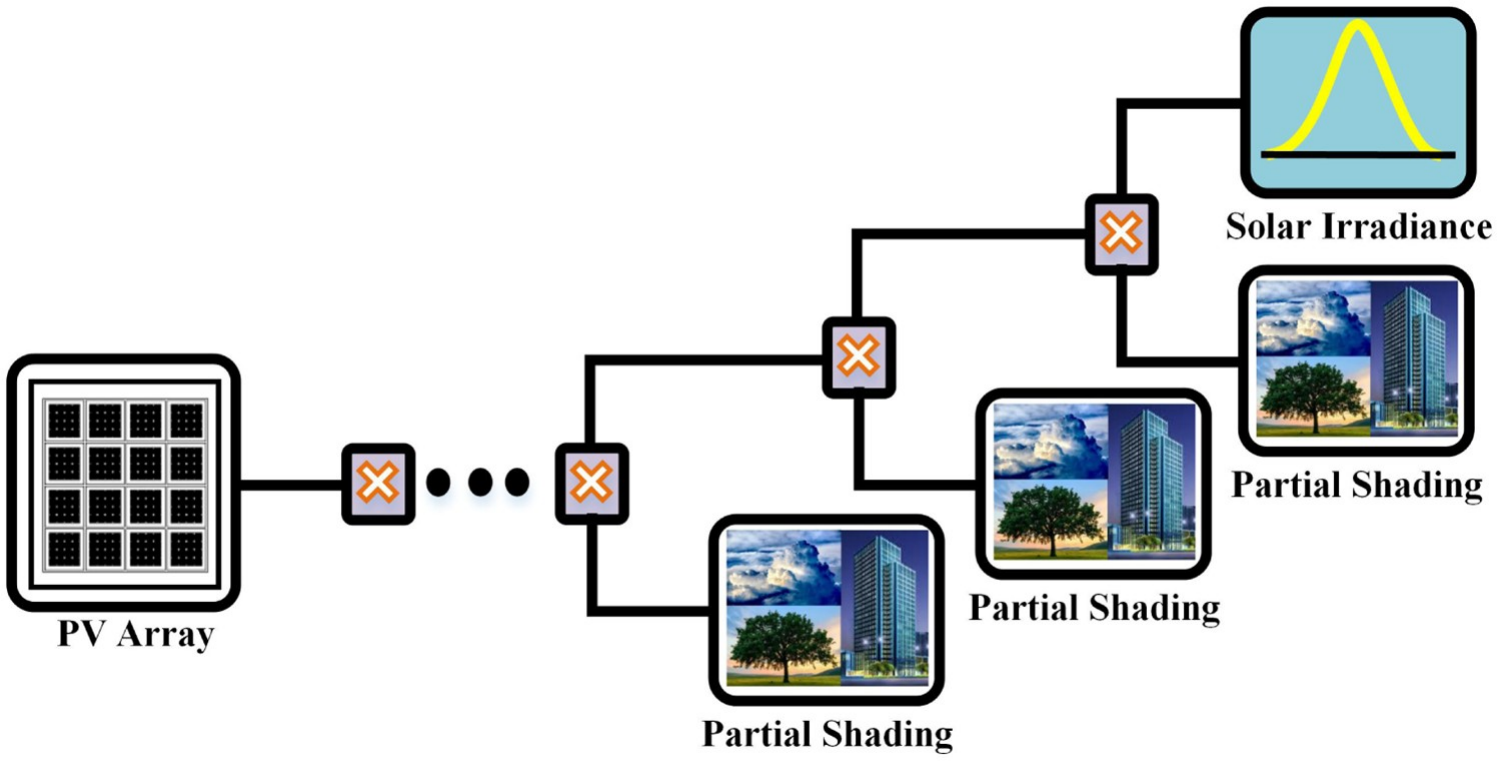
Introducing partial shading effect.

PSCs arise due to moving clouds, airplanes, dust, and shadows of the building. They cause a sudden drop in irradiation level as well as temperature ranging from a short interval of time to many hours. To evaluate the performance of proposed controllers for this type of phenomenon, PSCs are introduced in solar and temperature profiles by introducing multiplying factors at certain intervals of time as shown in Fig 17.

**Fig 18:**
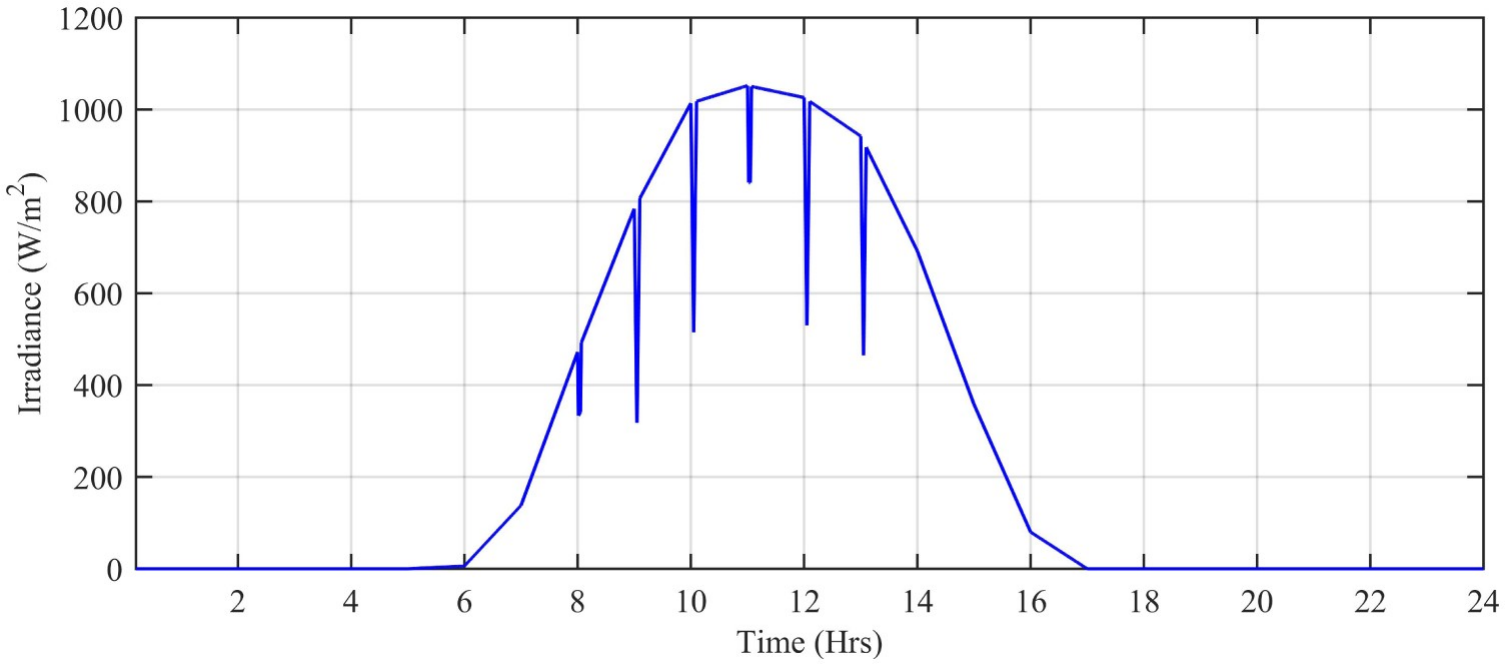
Partial shading profile of solar irradiance level (W/m^2^).

**Fig 19:**
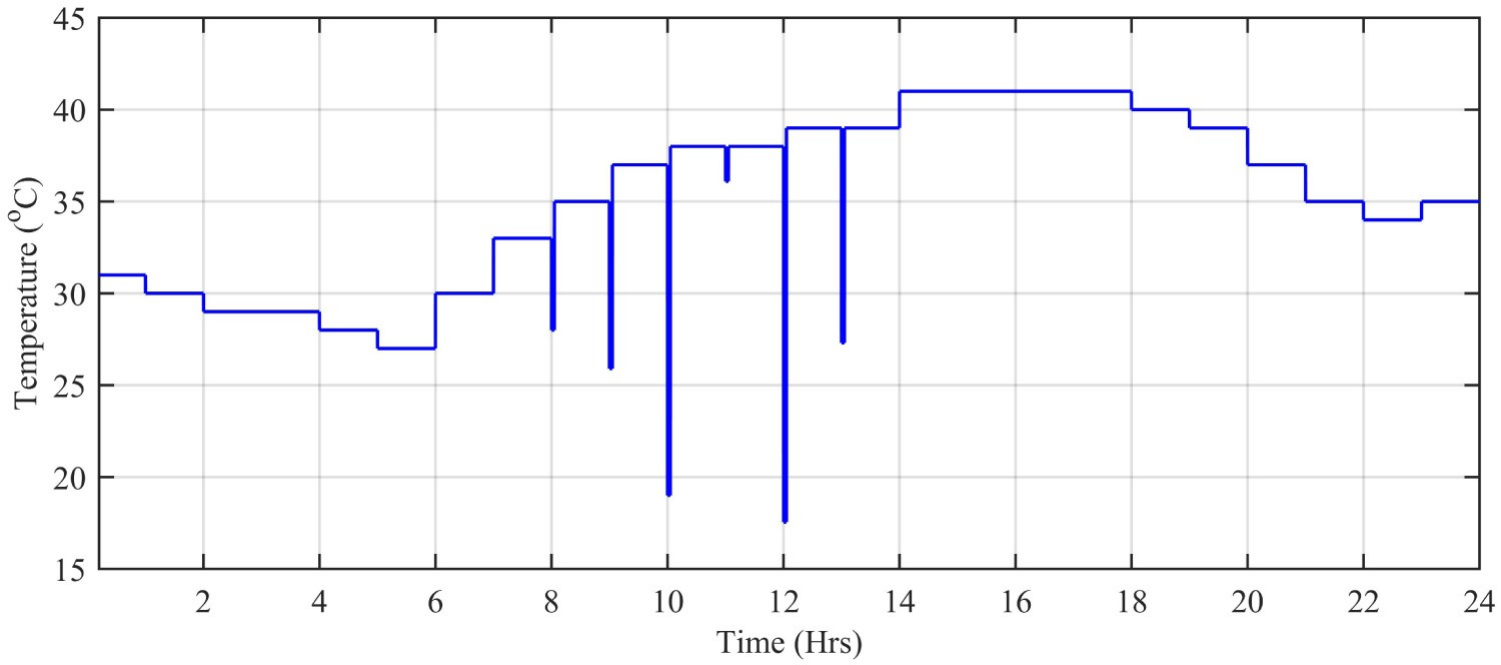
Partial Shading profile of ambient temperature (°C).

This suddenly changes the magnitude of irradiation level, as well as temperature, thus produce fluctuations in irradiation and temperature curve as shown in Figs 18 and 19.

**Fig 20:**
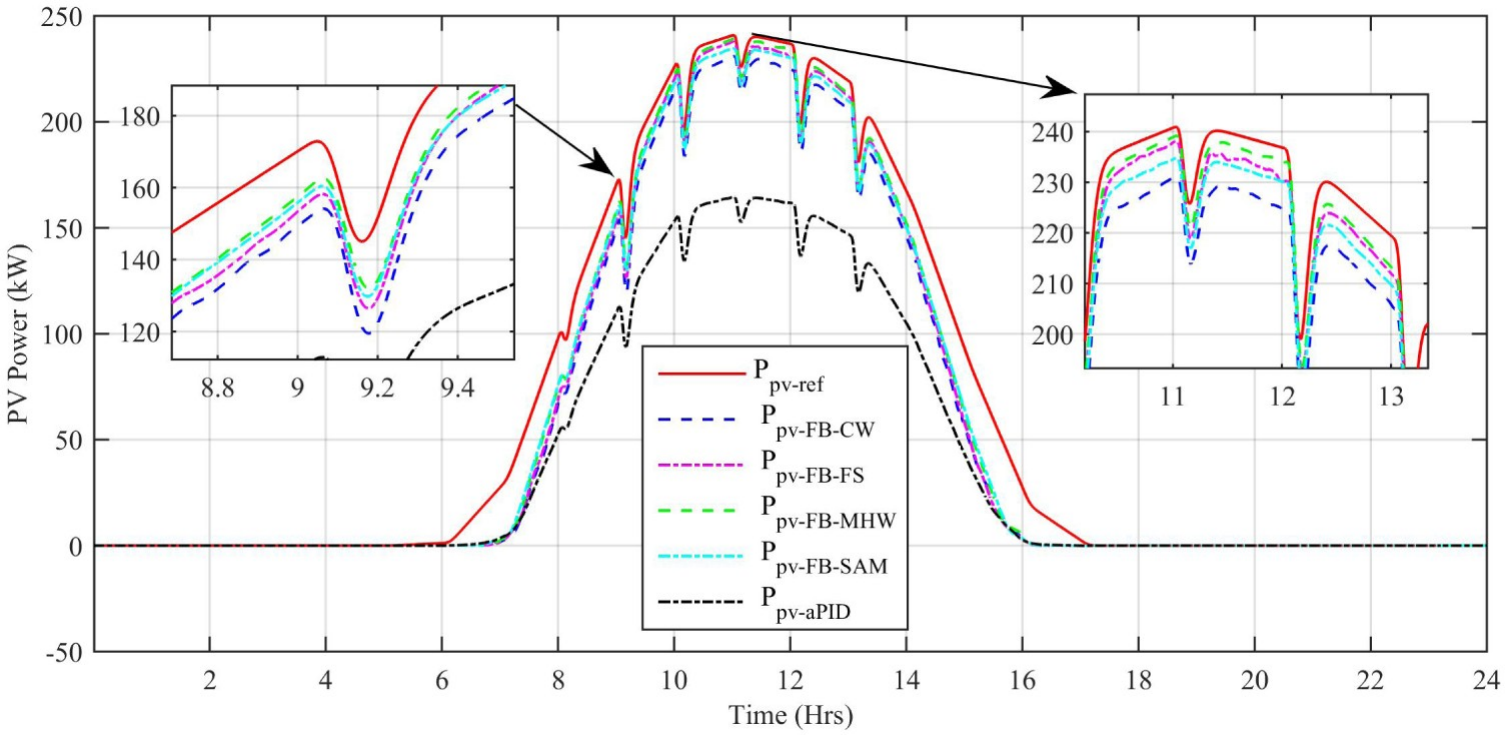
PV MPP tracked vs reference power for PSC profile.

Fig 20 shows the output power tracked by all the proposed control schemes compared with aPID. The sudden drops in irradiation and temperature level are successfully adopted by all the proposed control schemes, whereas, aPID produces the least accurate results. It is obvious to note that the output power produced using FRANF-CW, FRANF-SAM, and FRANF-FS is better than each other but the performance of FRANF-MHW is superior over all the other proposed control schemes. The maximum and average power errors of all the control schemes calculated according to Eq (57) are given in Table 4.

**Table 4:** Peak power error and average power error of all control schemes for partial shading condition.

Adaptive FBL based Control Scheme	Maximum Peak Power Error (*kW*)	Average Power Error (*kW*)
aPID	76.39	23.45
FRANF-CW	39.55	9.145
FRANF-SAM	35.79	7.225
FRANF-FS	40.64	7.562
FRANF-MHW	38.39	6.605

**Fig 21:**
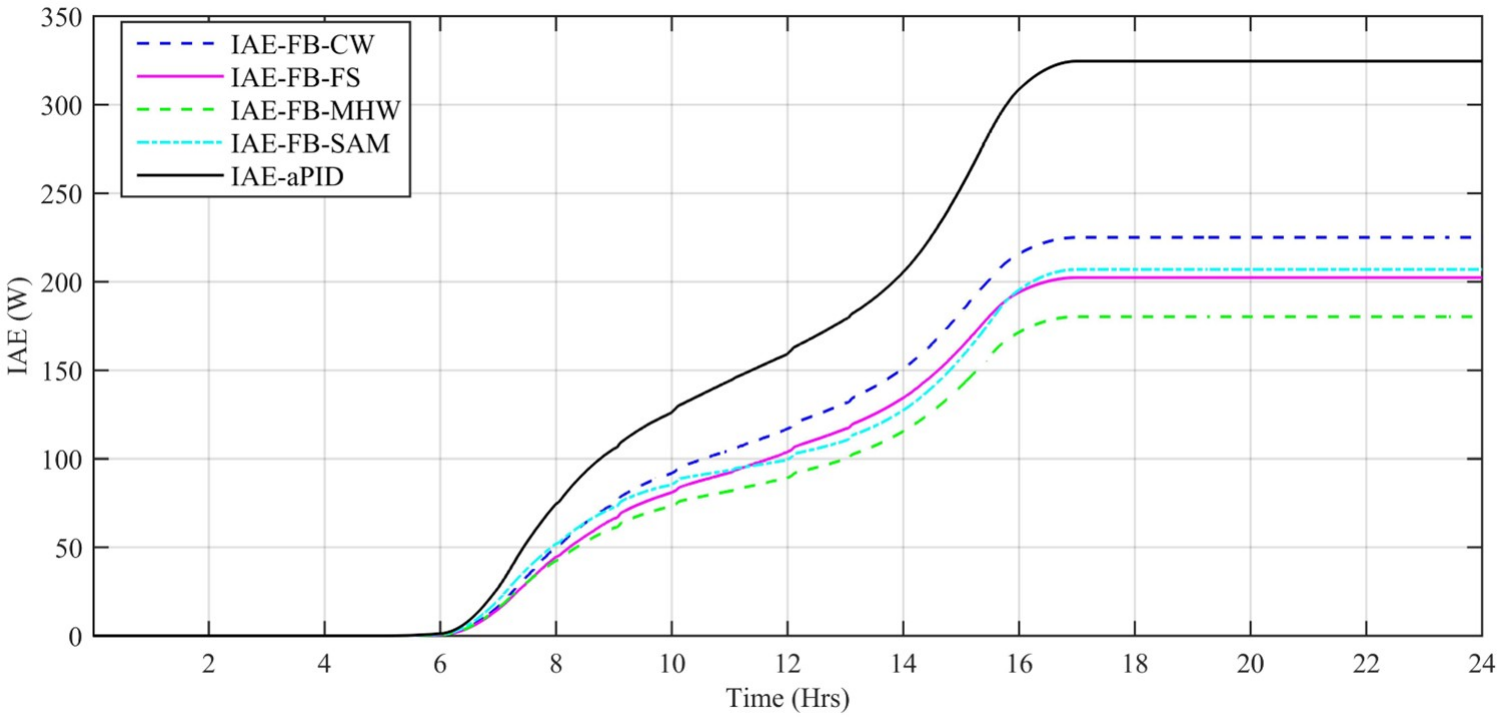
IAE for partial shading solar profile.

**Fig 22:**
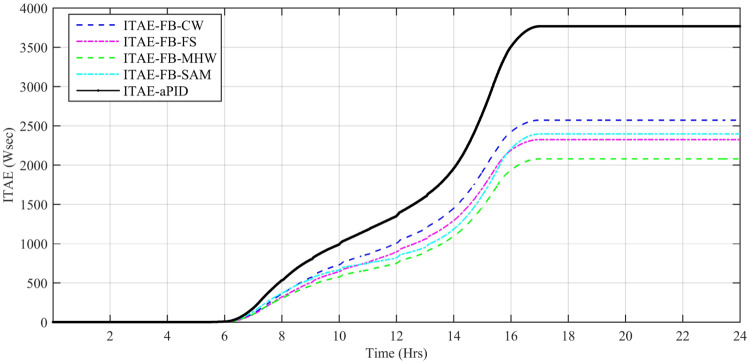
ITAE for partial shading solar profile.

**Fig 23:**
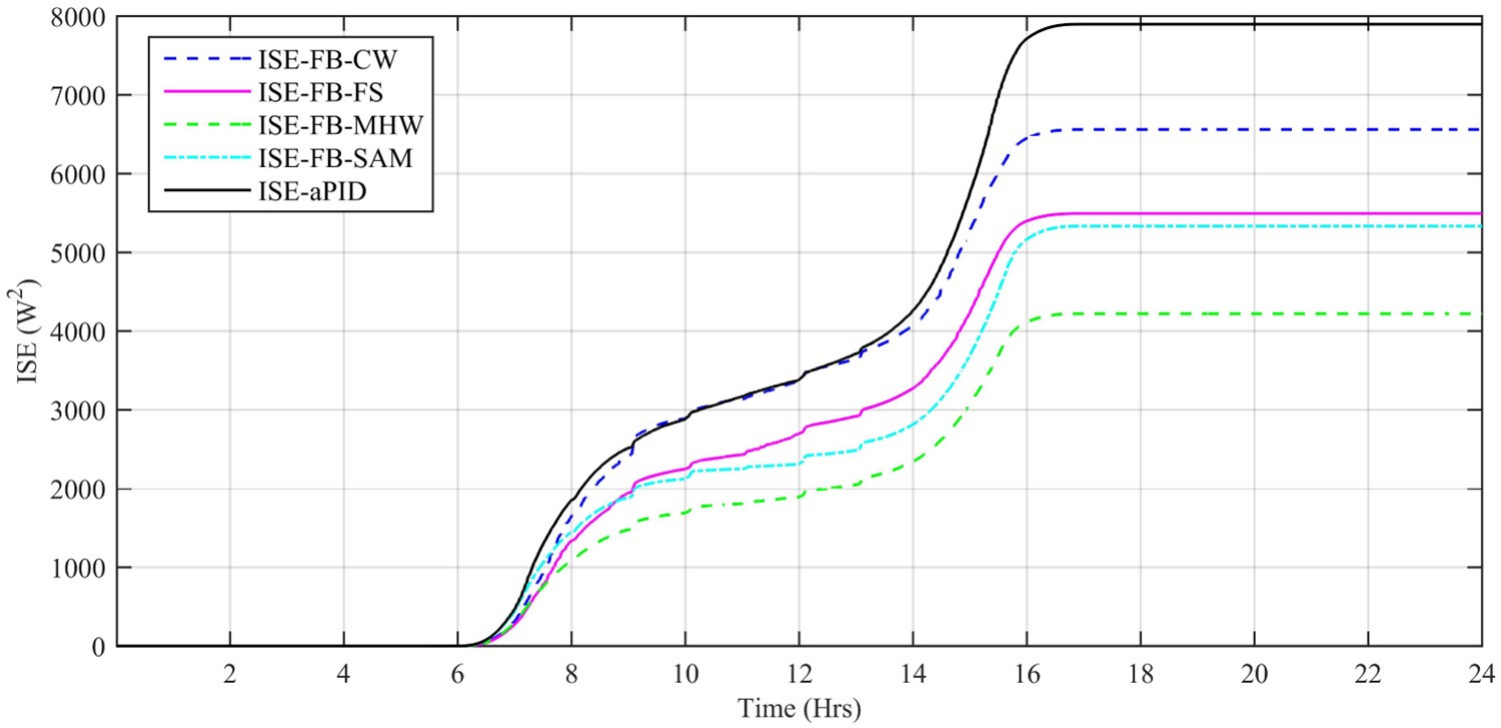
ISE for partial shading solar profile.

**Fig 24:**
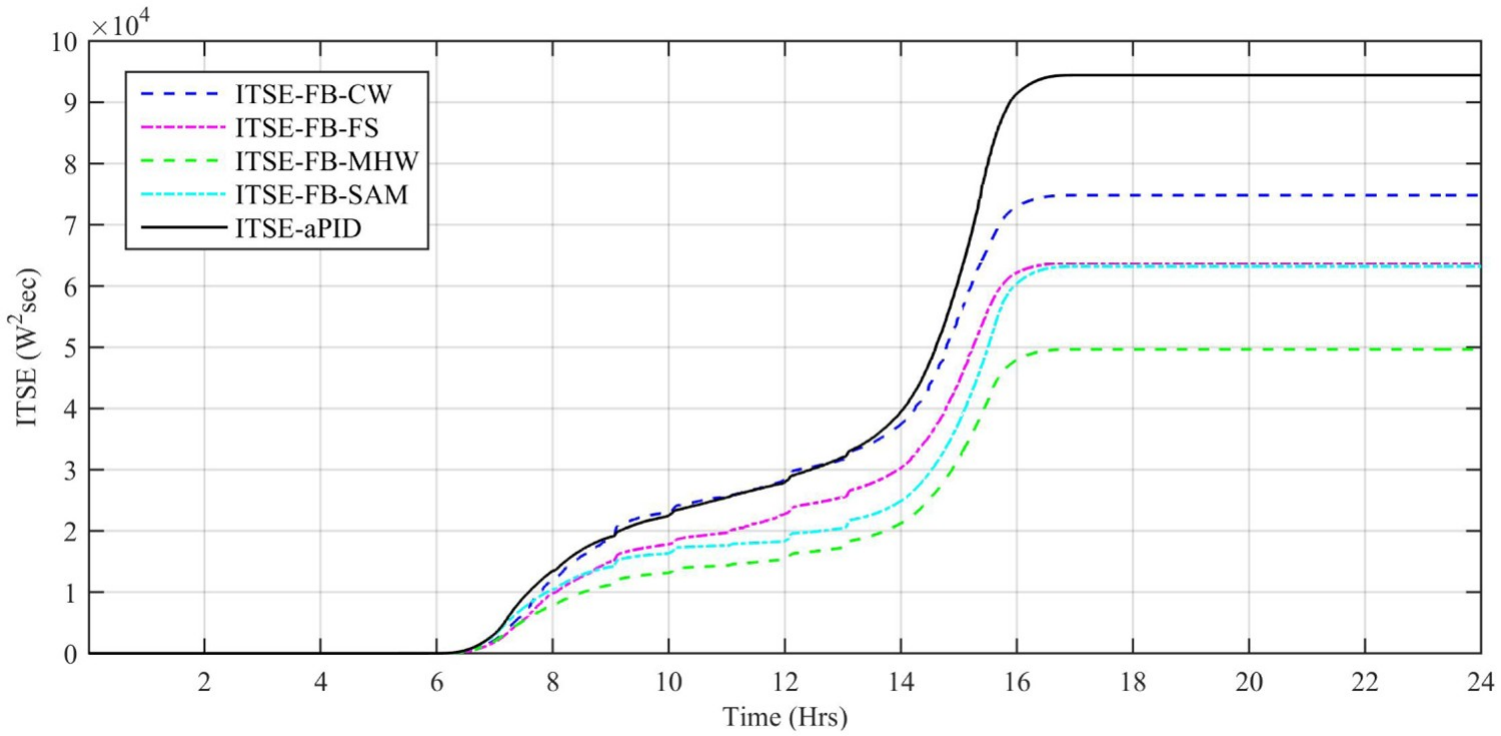
ITSE for partial shading solar profile.

Performance indexes calculated based on *P*_*error*_ in Eq (57) are shown in the Figs 21, 22, 23, and 24. Comparative analysis based on the various performance indexes is shown in Table 5.

**Table 5:** Performance indexes for step changing solar.

Adaptive FBL based Control Scheme	IAE (W)	ITAE (Wsec)	ISE (W^2^)	ITSE (W^2^sec)
aPID	324.6	3768	7899	94430
FRANF-CW	225	2573	6560	74820
FRANF-FS	202.4	2325	5494	63750
FRANF-SAM	206.9	2396	5333	63170
FRANF-MHW	180.3	2079	4221	49660

**Fig 25:**
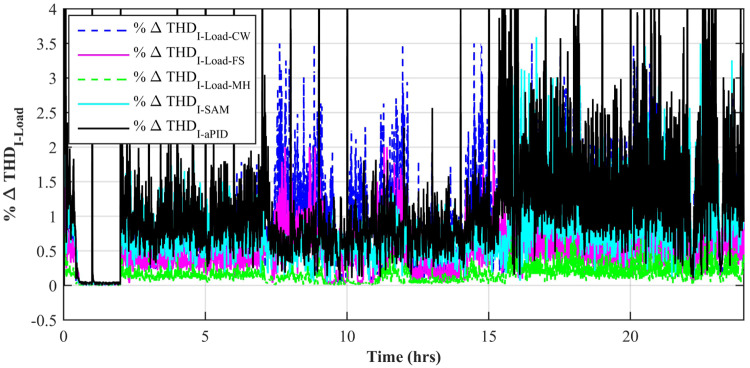
%age THD change in load current for partial shading solar profile.

**Fig 26:**
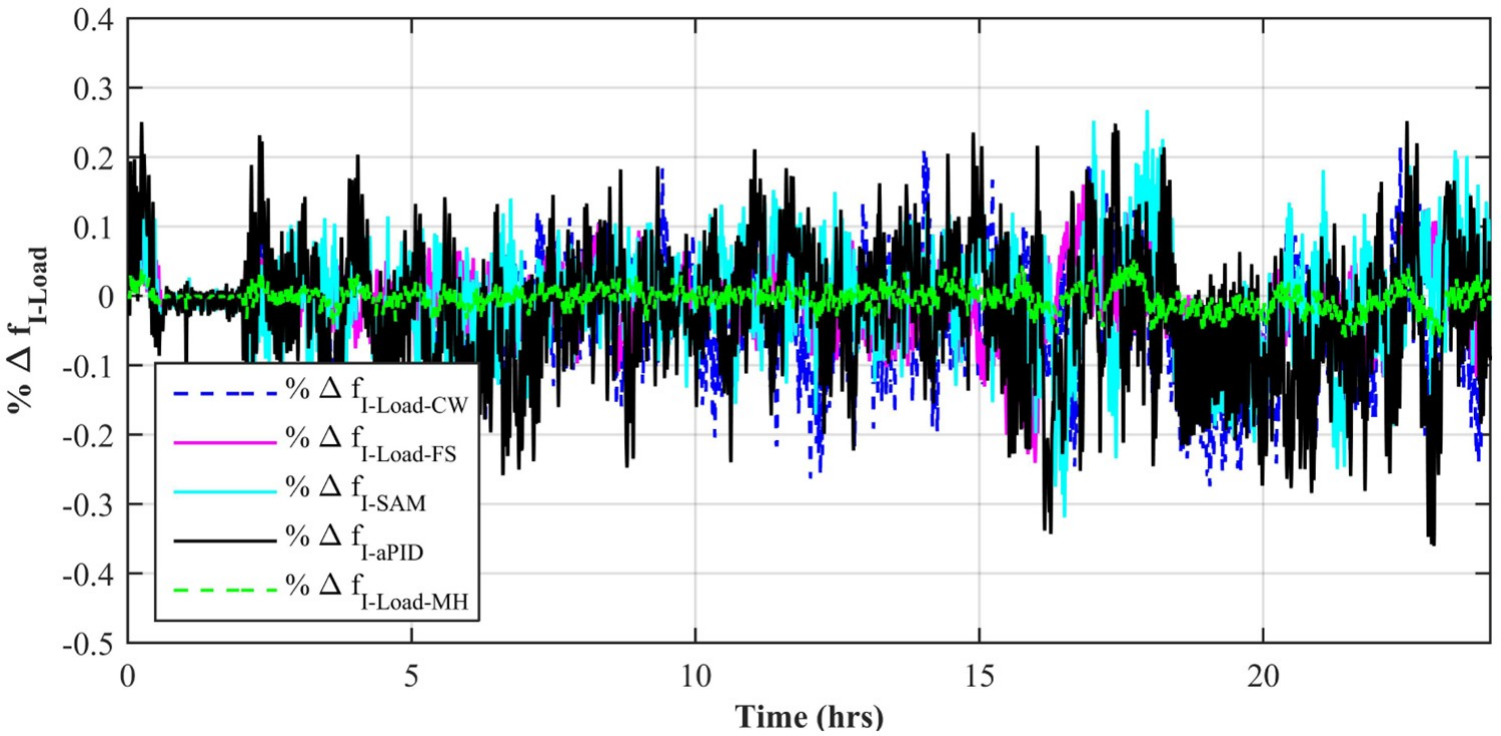
%age frequency change for partial shading solar profile.

Fig 25 shows a comparison of the percentage change in total harmonic distortion (THD) for load current for all control schemes. It is obvious to note that the percentage change in THD due to the adaptive FBL embedded FRANF-MHW control scheme is the smallest compared to the rest of the proposed controllers. The percentage change in frequency is also shown in Fig 26 for all proposed controllers. It can be observed that the percentage change in frequency due to adaptive FBL embedded FRANF-MHW control scheme is the least among all the proposed schemes.

#### 3.1.3 Daily field data of solar irradiation and temperature in Islamabad

**Fig 27:**
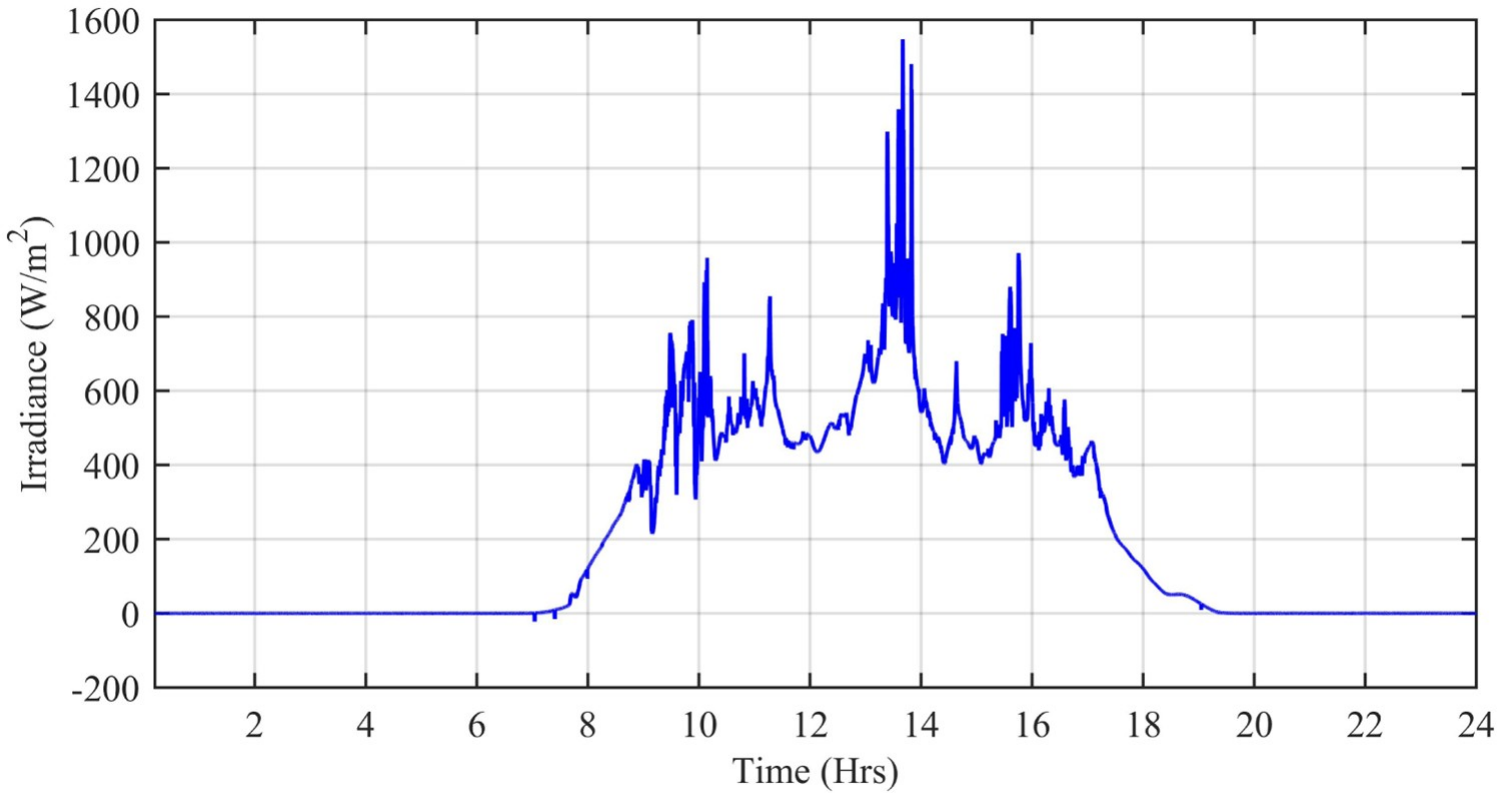
Solar irradiance level (W/*m*^2^.

**Fig 28:**
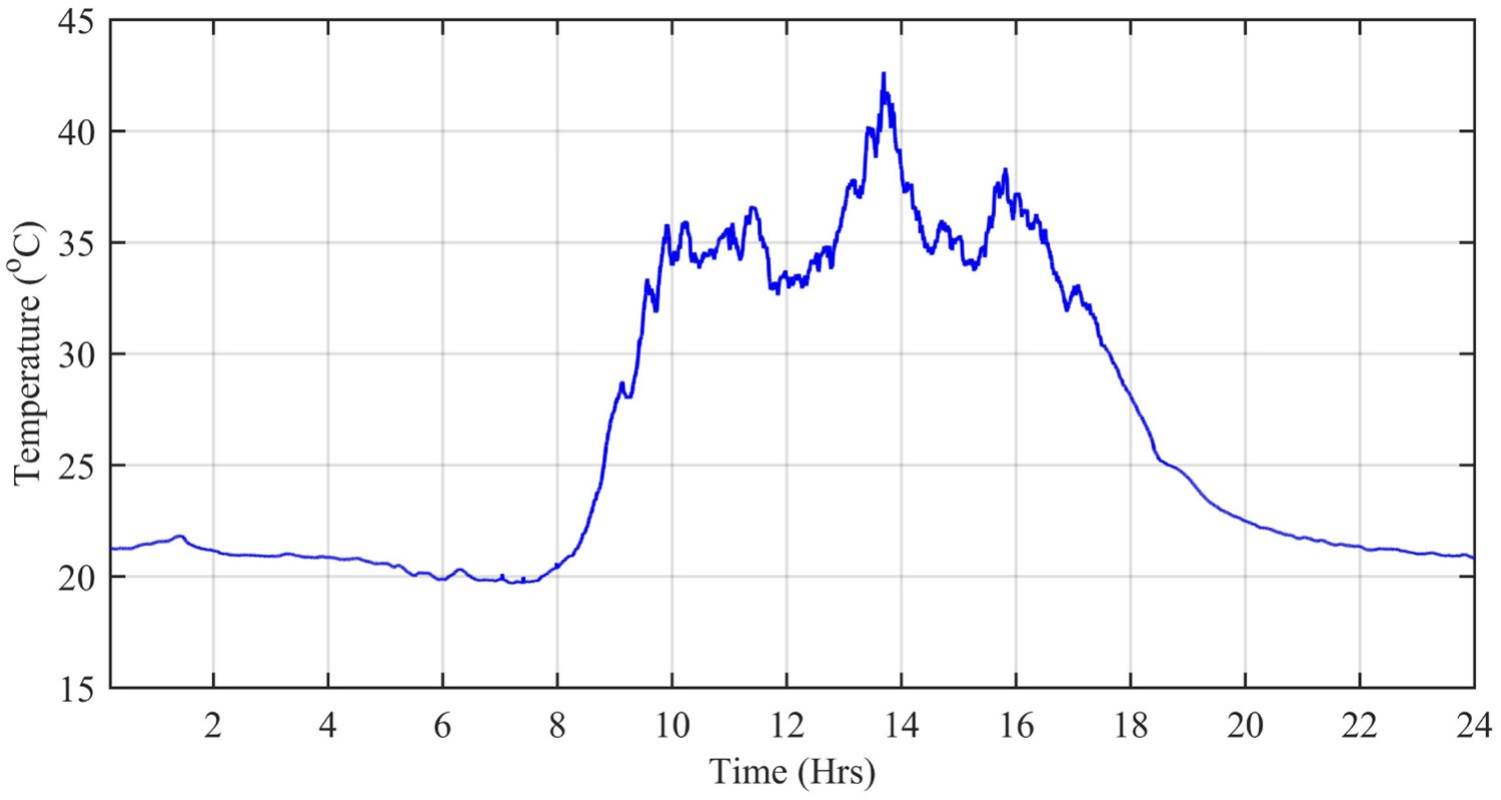
Ambient temperature level (°C).

The wind speed (*m*/*s*), ambient temperature (°C), and solar irradiation (W/*m*^2^) are obtained from the Pakistan Meteorological Department (PMD) for a complete solar day at Islamabad station. In this simulation, each hour is modeled for one second of simulation time. The case study taken is the Defense Housing Authority (DHA), Islamabad, Pakistan. The irradiation varies with the appearance of the sun. The average irradiation level during day time is about 1000 W/*m*^2^, while the average temperature level is 20°*C* having a maximum peak of about 42.6°C during day time. Fig 27 shows the irradiance profile and Fig 28 shows the temperature profile used for this case study.

**Fig 29:**
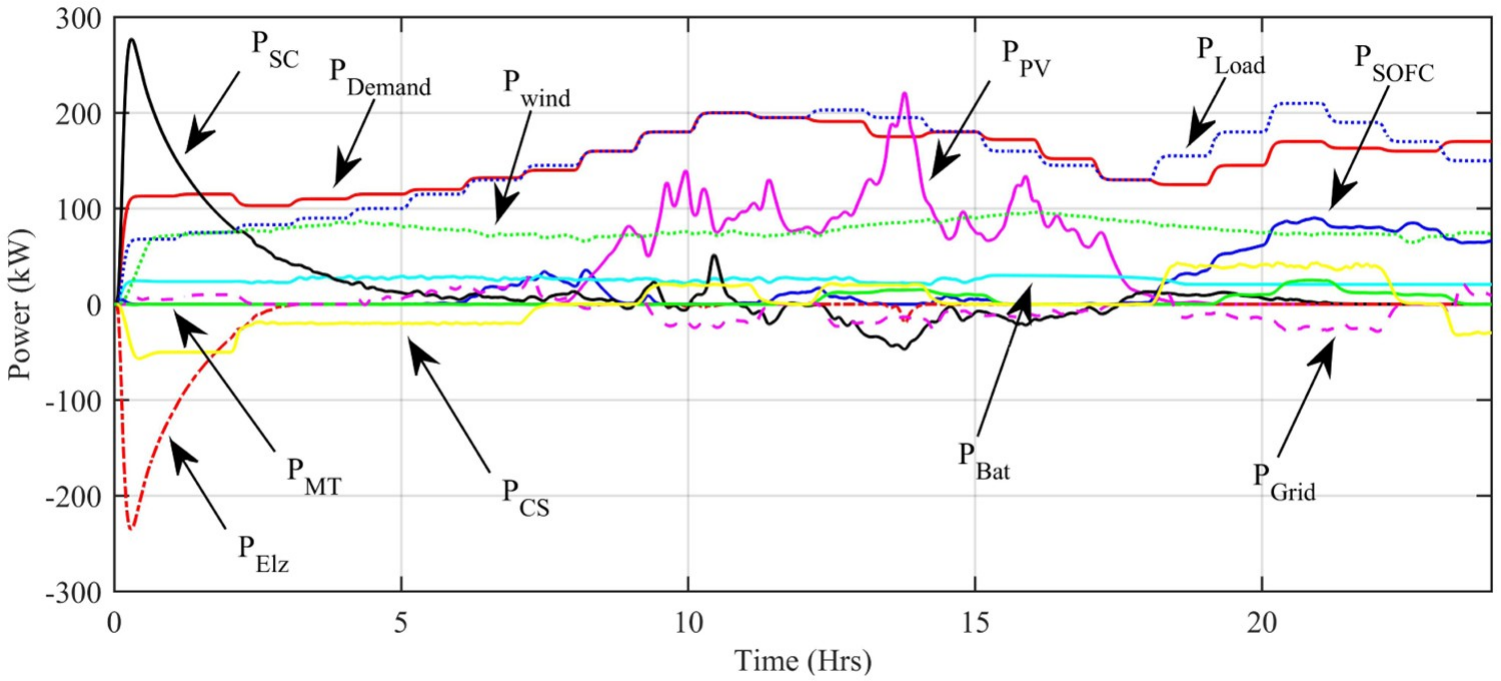
Generated power of all sources and load power.

**Fig 30:**
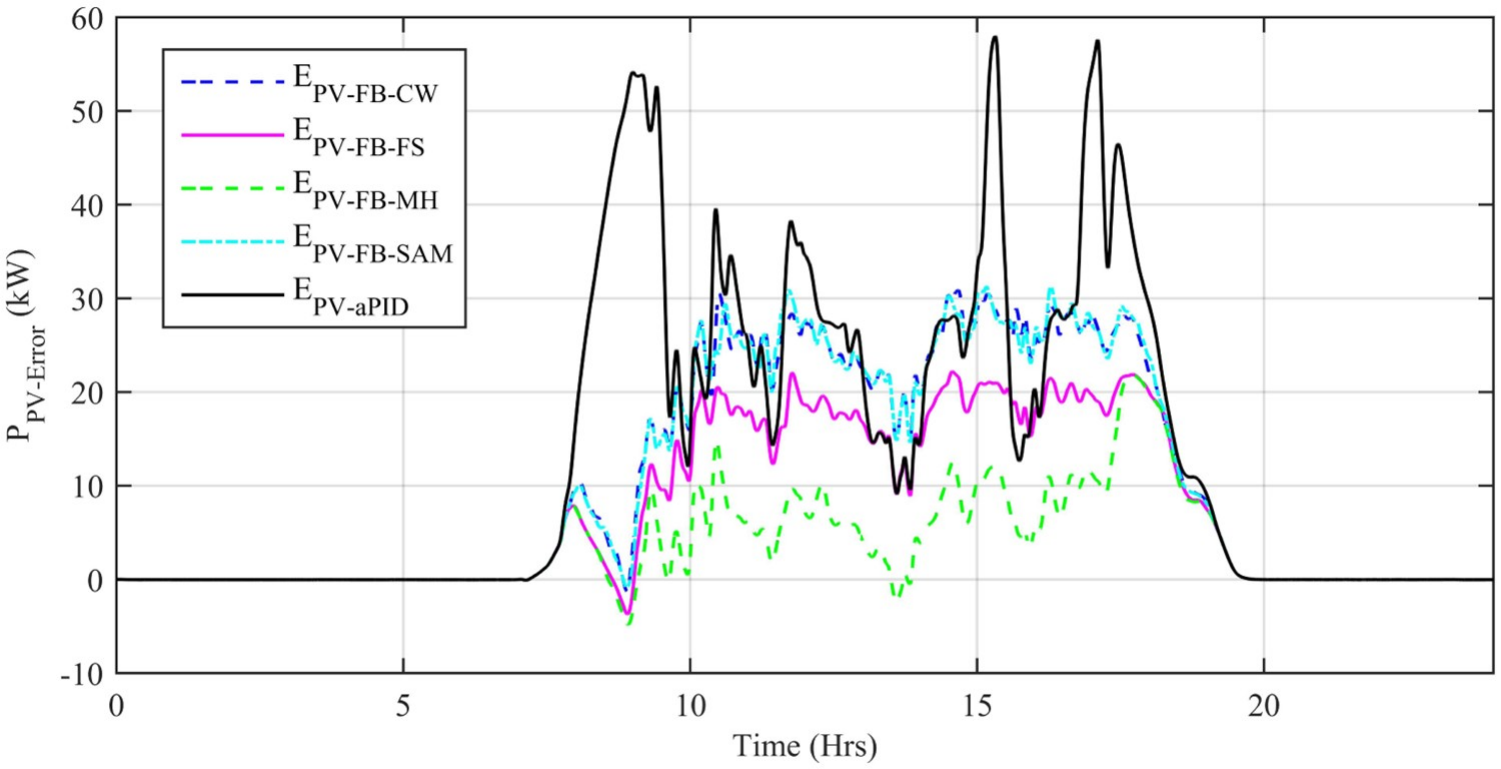
Power error in MPPT.

Fig 29 shows the complete power profile of all the sources and total load connected in the microgrid system during one day. Fig 30 shows the comparison of the power error of all the proposed controllers with aPID. The sudden spikes in the results are because of sudden changes in the irradiance level under PSCs that causes a control error. This initiates the controller which adjusts the duty cycle to minimize the power error.

Fig 30 also shows that the maximum power error generated by the least performing controller adaptive FBL embedded FRANF-SAM is not exceeding 31.3 kW at its highest peak, whereas the maximum power error generated by the best performing controller i.e. Adaptive FBL embedded FRANF-MHW is below 21.9 kW at its highest peak. However, aPID produced a maximum power error of 58kW at its highest peak. The maximum peak and average power error of all the control schemes starting from the least performance to the best is shown in Table 6.

**Table 6:** Peak power error and average power error of all proposed controllers.

Adaptive FBL based Control Scheme	Maximum Peak Power Error (*kW*)	Average Power Error (*kW*)
aPID	58	26.456
FRANF-SAM	31.3	10.0861
FRANF-CW	30.9	10.0889
FRANF-FS	22.3	7.264
FRANF-MHW	21.9	3.5917

**Fig 31:**
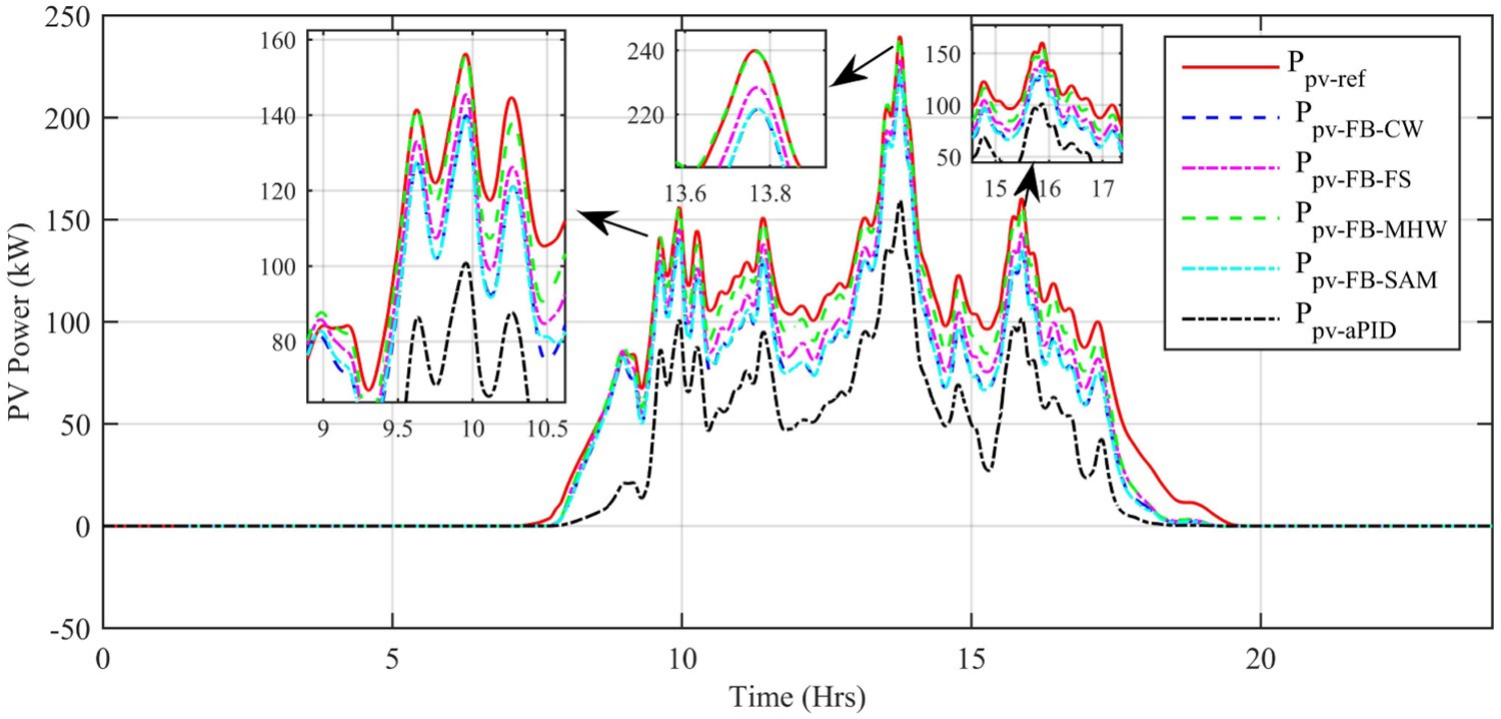
PV MPP tracked vs reference power.

The output power comparison of all proposed controllers and reference power is shown in Fig 31. For every sharp change in reference power, there is also a sharp spike in tracked power at the same instant of time. This proves the ability of proposed controllers in dealing with sharp sudden changes in the reference signal. The only difference in the performance of the proposed controllers is the net output generated power at the same instant of time. It can be seen clearly that adaptive FBL embedded FRANF-SAM and adaptive FBL embedded FRANF-CW are producing low output power as compared to adaptive FBL embedded FRANF-FS and Adaptive FBL embedded FRANF-MHW. This is because of the different consequent structures used in FRANF. The *P*_*PV*−*FB*−*MHW*_ acquires the PV system output power with steady-state error = 0.3 kW, undershoot = -9.19% and overshoot = 4.39%. The *P*_*PV*−*FB*−*FS*_ extracts the PV system output power with steady-state error = 11.5 kW, undershoot = -20.95% and overshoot = 2.48%. The *P*_*PV*−*FB*−*SAM*_ obtained the PV system output power with steady state-error = 18.1 kW, undershoot = -26.99% and overshoot = 1.16%. The *P*_*PV*−*FB*−*CW*_ obtained the PV system output power with steady state-error = 18.4 kW, undershoot = -28.33% and overshoot = 1.49%.

**Fig 32:**
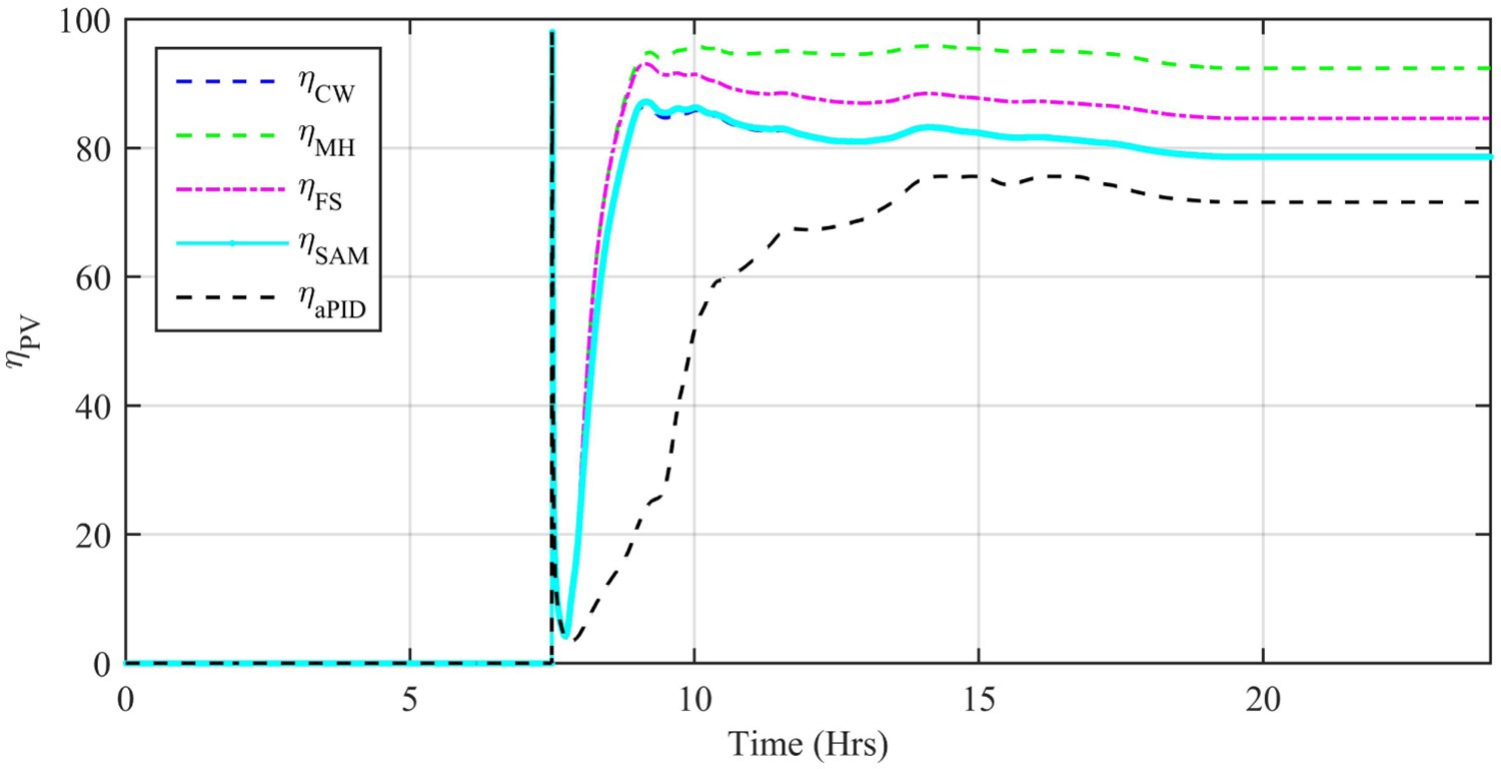
Efficiency.

The dynamic efficiency *η*_*PV*_ of various controllers on the basis of their tracked powers and power error are calculated as:

$$\begin{array}{c} \eta_{PV}=\frac{\int_{t_{o}}^{t_{f}}P_{PV}dt}{\int_{t_{o}}^{t_{f}}P_{ref}dt}\times 100\% \end{array}$$ (58)

where; *P*_*PV*_ = *V*_*PV*_ × *I*_*PV*_, *t*_*o*_ = 0 *h* and *t*_*f*_ = 24 *h* are initial and final intervals respectively. The dynamic efficiency, *η*_*PV*_ of the proposed controllers are shown in Fig 32. The peak *η*_*PV*_ of aPID is 75.56%, adaptive FBL embedded FRANF-SAM is 87.1%, adaptive FBL embedded FRANF-CW is 86.4%, adaptive FBL embedded FRANF-FS is 93.1% and adaptive FBL embedded FRANF-MHW is 95.9%. The efficiency plot of all the controllers shows a small variation. This is due to continuous change in the reference signal according to the solar irradiation at that instant of time and shows the high sensitivity of the proposed controllers. The peak and average efficiency of all controllers starting from the least to the most efficient are shown in Table 7.

**Table 7:** Peak and average efficiency of all proposed controllers.

Adaptive FBL based Control Scheme	Maximum Peak Efficiency (%)	Average Efficiency (%)
aPID	75.56	62.65
FRANF-CW	86.4	77.71
FRANF-SAM	87.1	77.9
FRANF-FS	93.1	83.6
FRANF-MHW	95.9	90.2

**Fig 33:**
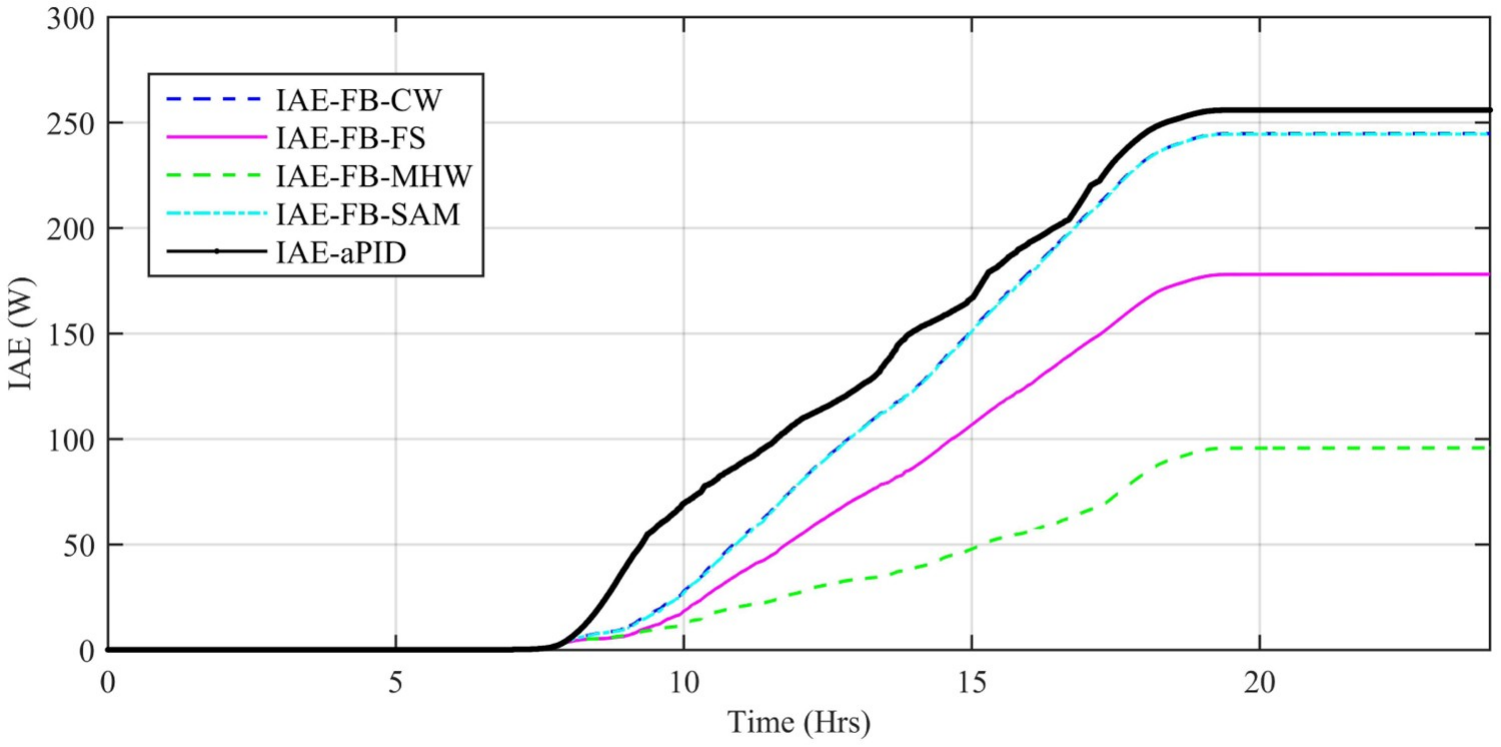
IAE.

**Fig 34:**
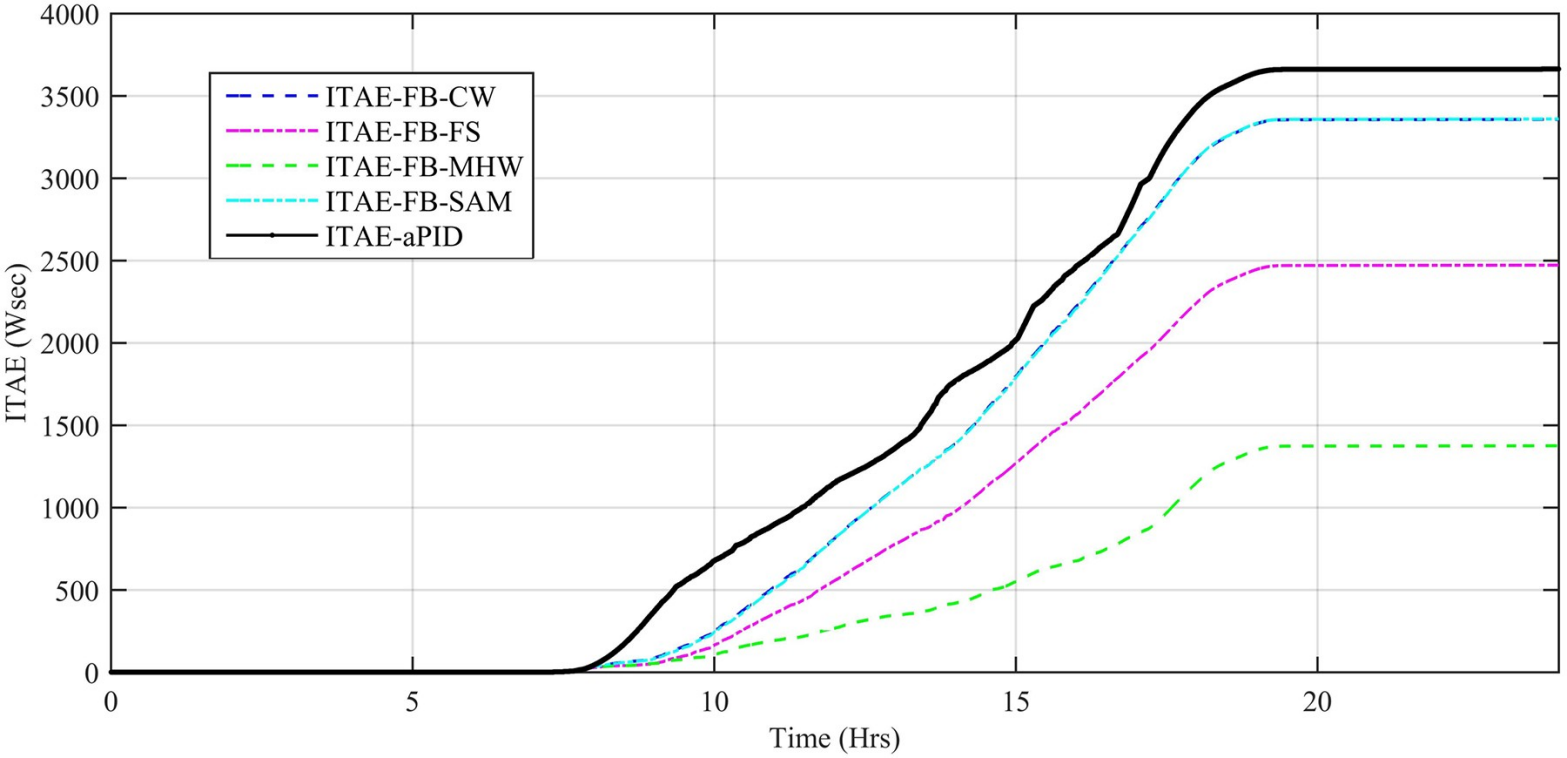
ITAE.

**Fig 35:**
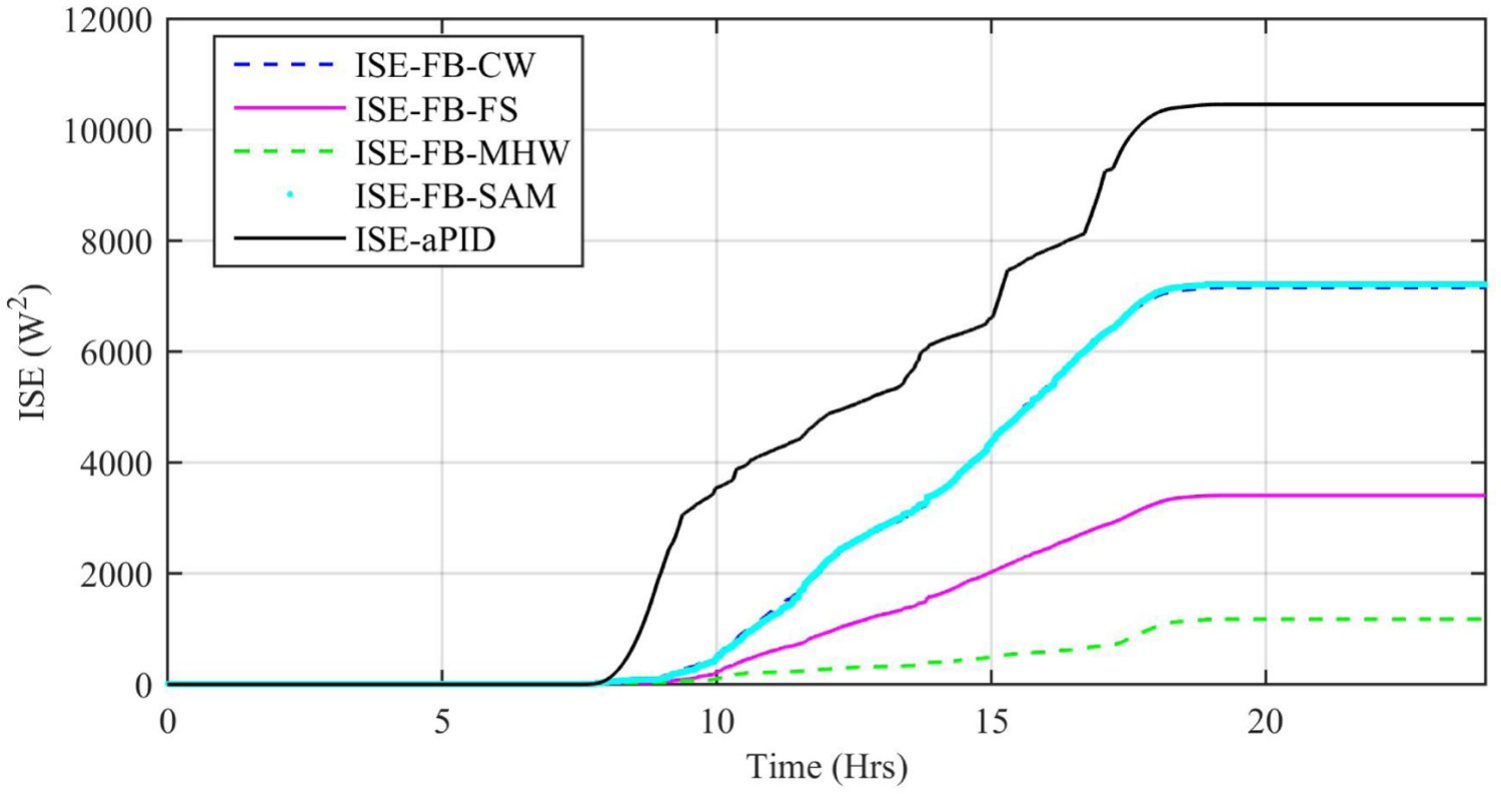
ISE.

**Fig 36:**
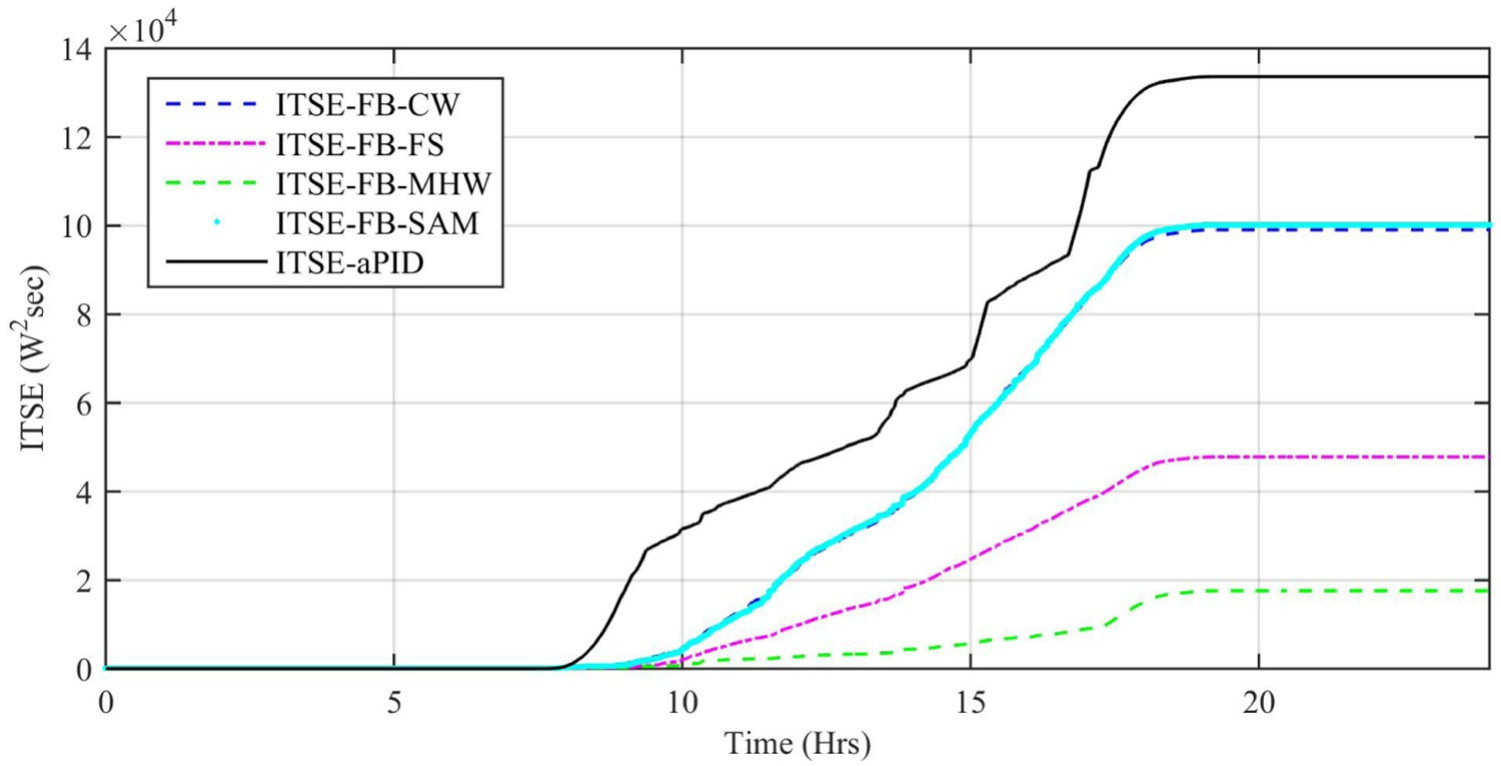
ITSE.

Performance indexes including Integral Square Error (ISE), Integral Time Square Error (ITSE), Integral Absolute Error (IAE), and Integral Time Absolute Error (ITAE), calculated based on *P*_*error*_ in Eq (57) as shown in Figs 33, 34, 35 and 36. The comparison of performance indexes plots shows that the accumulative error in all schemes increases with time. Again it is clear that the index of adaptive FBL embedded FRANF-MHW is least among all proposed controllers. Table 8 shows the values of various indexes of all the proposed controllers in contrast with aPID showing least to most performing from top to bottom.

**Table 8:** Performance indexes of all proposed controllers.

Adaptive FBL based Control Scheme	IAE (W)	ITAE (Wsec)	ISE (W^2^)	ITSE (W^2^sec)
aPID	255.9	3661	10460	133600
FRANF-SAM	244.4	3359	7213	100100
FRANF-CW	244.8	3356	7156	99070
FRANF-FS	177.9	2471	3408	47830
FRANF-MHW	95.7	1373	1175	17590

**Fig 37:**
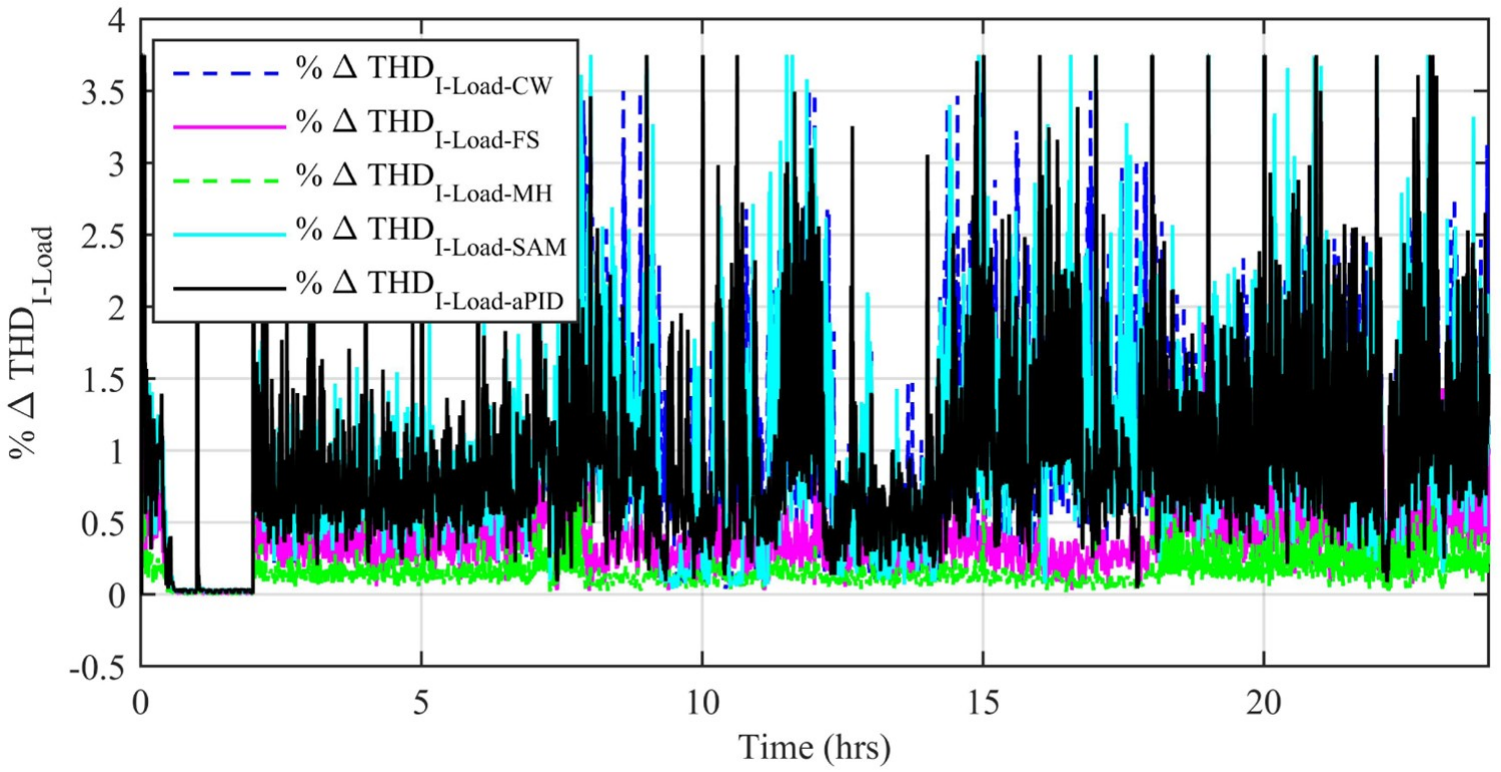
%age THD change in load current.

**Fig 38:**
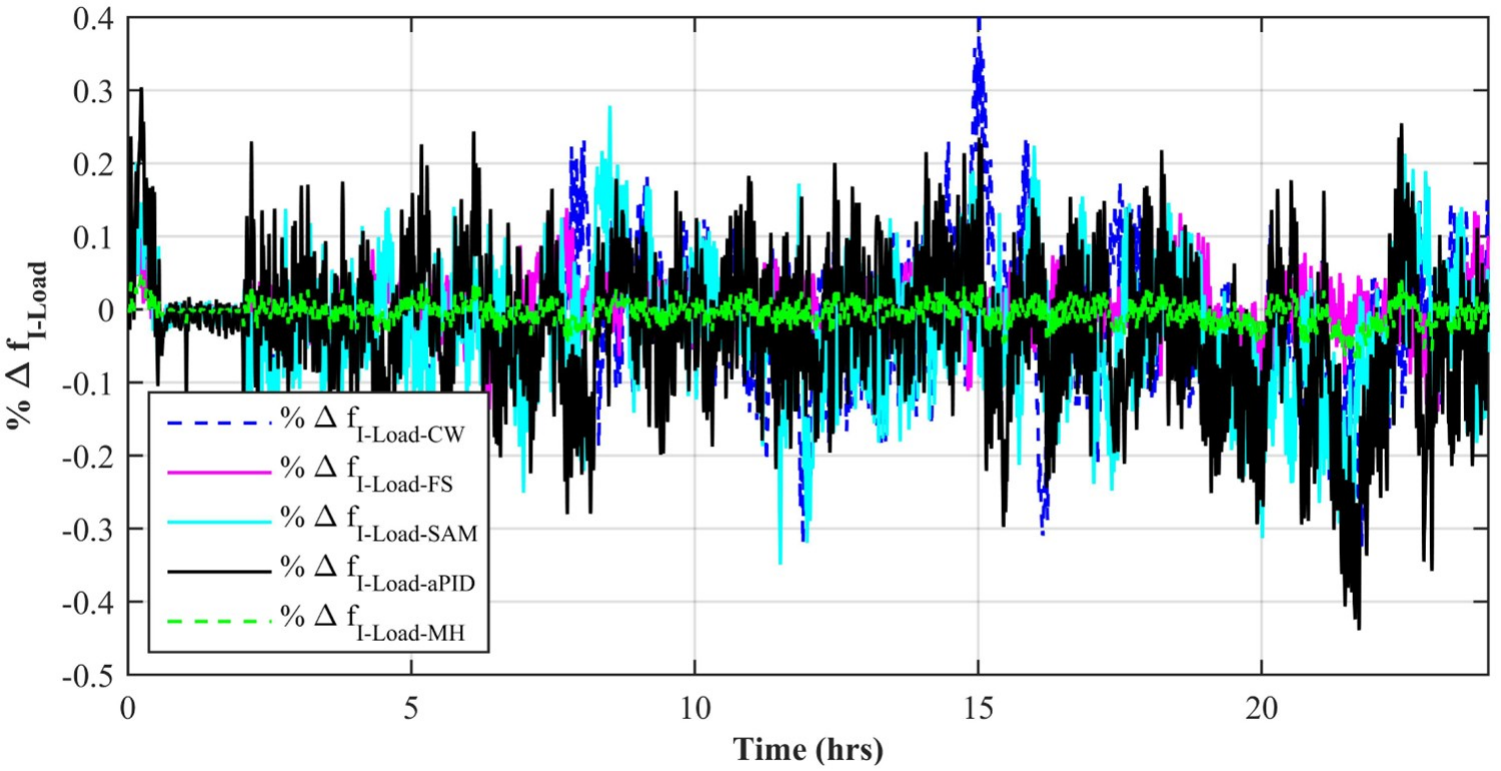
%age frequency change.

Fig 37 shows a comparison of the percentage change in total harmonic distortion (THD) for load current due to individual control scheme. The percentage change in THD complies with the IEEE standard 1547 [45]. The result shows that the percentage change in THD due to the adaptive FBL embedded FRANF-MHW control scheme is the smallest of all and proves its better performance among other proposed controllers. The percentage change in frequency is also shown in Fig 38 for all proposed controllers. It can be observed that the percentage change in frequency due to the adaptive FBL embedded FRANF-MHW control scheme is almost flat and nearly zero. The percentage change in frequency for all the proposed controllers is within the acceptable range according to the IEEE standard 1547 [45].

**Fig 39:**
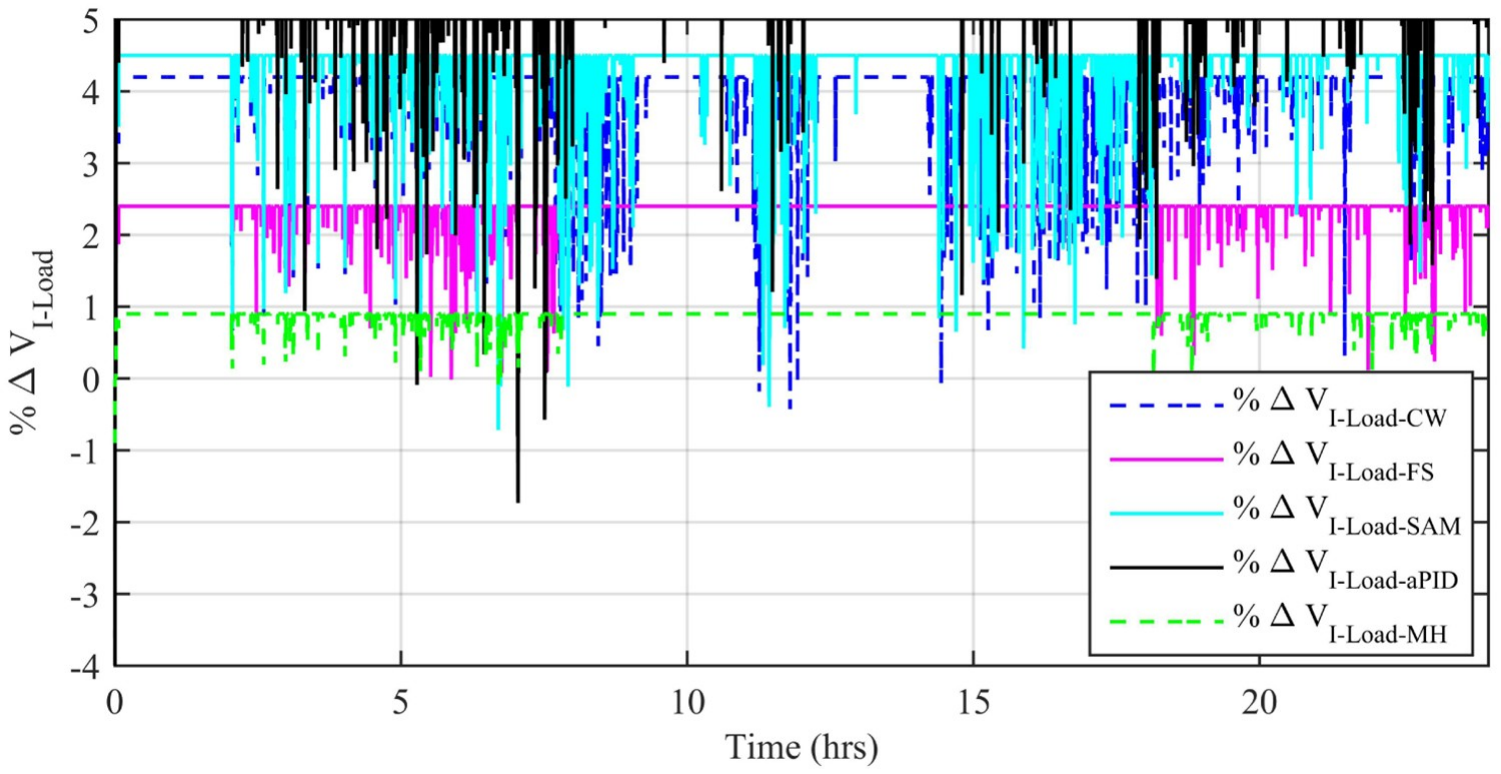
%age *V*_*RMS*_ change.

**Fig 40:**
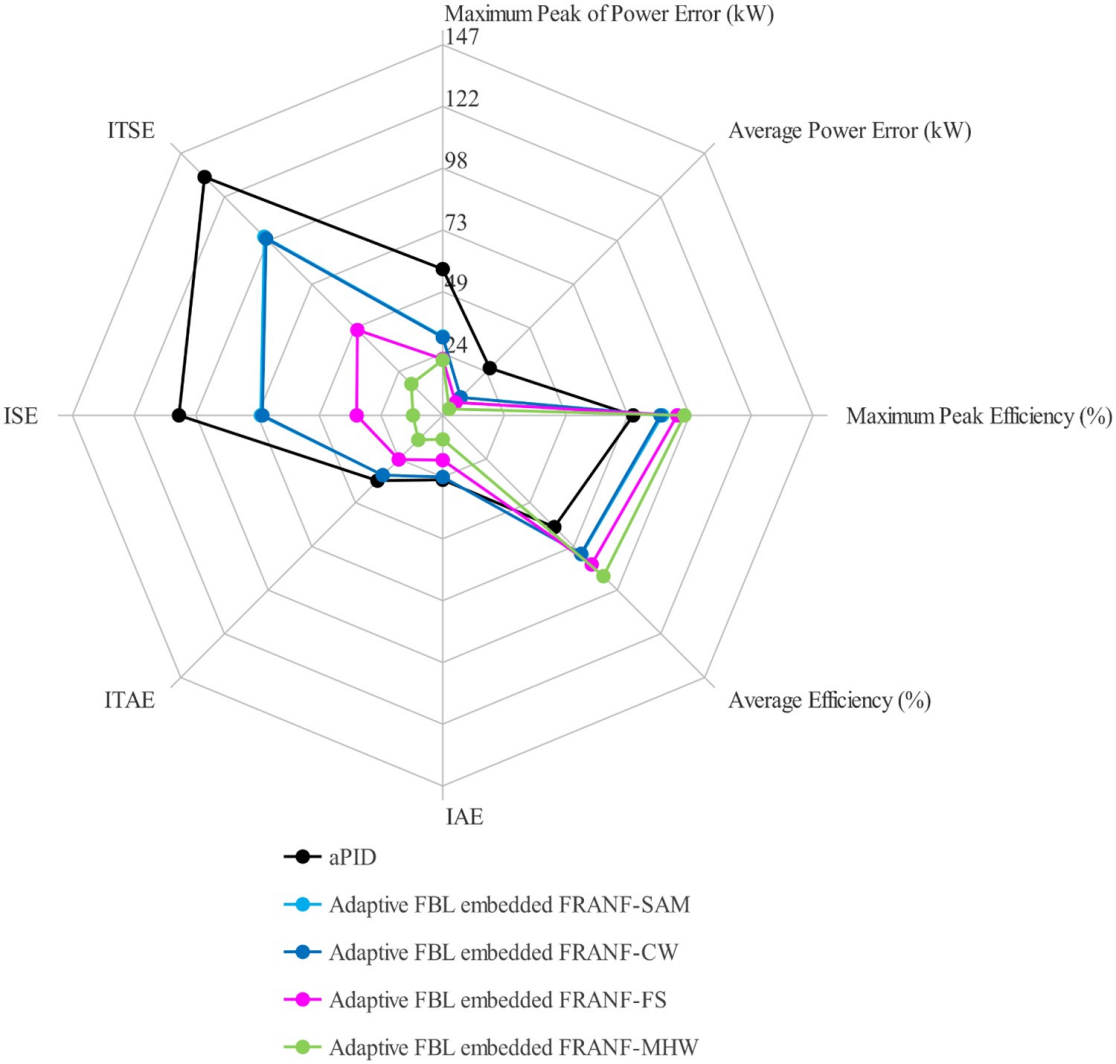
Spider Chart of all parameters.

Another factor of comparison is the percentage change in voltage (*V*_*RMS*_) produced in the AC-bus during the conversion process. Fig 39 shows the performance of the individual proposed scheme. It can be seen that the percentage change in *V*_*RMS*_ due to adaptive FBL embedded FRANF-MHW control scheme has the least value among all proposed control scheme and thus ensures its superior performance. The overall performance of all the described controllers is compared based on power quality, average and peak power error in MPPT, efficiency, performance indexes, percentage THD change, percentage frequency change, and percentage *V*_*RMS*_ in load current. For daily field data, a spider chart is plotted for all comparable performance parameters in Fig 40. Statistical values are scaled to a specific level for each individual parameter to obtain a clear view. The graphical view of IAE, ITAE, ISE, ITSE, maximum peak power error, and average power error in Fig 40 shows that adaptive FBL embedded FRANF-MHW control has the least values for these parameters as compared to other proposed controllers. Whereas, maximum peak efficiency and average efficiency values of Adaptive FBL embedded FRANF-MHW are the highest comparably. The spider chart shows the superior performance of the adaptive FBL embedded FRANF-MHW control scheme over other proposed controllers in a single glance.

## Conclusions

In this article, four intelligent control schemes are presented for the MPPT problem of a grid-connected PV subsystem in an SMG-HPS for three different solar and temperature profiles. Results of all the control schemes are compared against one another and aPID for various parameters obtained through simulations. The performance of all the proposed control schemes is within the acceptable range and simply cannot be rejected at any ground. However, the overall analysis shows the performance of the adaptive FBL embedded FRANF-MHW is superior to all the other proposed control schemes. This is due to the use of a continuous signal wavelet that is the MHW in the consequent part of the identifier as well as the adaptive recurrent weights in antecedent and consequent parts of the same identifier. On the other hand, the CW is a discrete composite mathematical function and the recurrent weight of the consequent part of this and other proposed identifier schemes are fixed gains that are not being updated during the simulation.

Future studies include testing of adaptive FBL embedded FRANF hybrid wavelet control compared to those presented in this article.

## Supporting information

S1 File (PDF)
