# Supplementary material for: Nonlinear adaptive NeuroFuzzy feedback linearization based MPPT control schemes for photovoltaic system in microgrid
Source: PLoS One. 2020 Jun 30;15(6):e0234992. doi: 10.1371/journal.pone.0234992 (PMC7326197; doi:10.1371/journal.pone.0234992)
Supplement: S1 File — (PDF) [file pone.0234992.s001.pdf]

# Nomenclature

| Variables | | Abbreviations | |
|---|---|---|---|
| $v_{pv}$ | output voltage of PV | FBL | feedback linearization |
| $i_{pv}$ | output current of PV | FRANF | full recurrent adaptive neuroFuzzy |
| $i_p$ | cell's photocurrent | SMG | smart microgrid |
| $i_D$ | diode's photocurrent | HPS | hybrid power system |
| $i_s$ | cell's reverse saturation current | PSC | partial shading condition |
| $i_{RS}$ | d-q components of the grid current | PV | Photovoltaic |
| $T_c$ | cell's absolute working temperature (K) | P&O | perturb and observe |
| $T_{ref}$ | cell's reference temperature (K) | OCV | open circuit voltage |
| $Z$ | total solar irrdiation ($W/m^2$) | HCA | hill climbing algorithm |
| $e_g$ | energy band gap of semiconductor used in cell | IC | incremental conductance |
| $n_p$ | number of parallel panels | RC | ripple correlation |
| $n_s$ | number of series panels | ANN | artificial neural network |
| **Control Schemes Parameters** | | MPP | maximum power point |
| $m_{ij}$ | mean of $i$th input and $j$th membership function | MPPT | maximum power point tracking |
| $\sigma_{ij}$ | variance of $i$th input and $j$th membership function | HVDC | high voltage direct current |
| $c_k$ | centroid of then-part | aPID | adaptive PID |
| $V_k$ | finite positive volume | WT | wind turbine |
| $d_{ij}$ | dilation of wavelet | MT | micro-turbine |
| $t_{ij}$ | translation of wavelet | SOFC | solid oxide fuel cell |
| $\mu_{ij}$ | membership function | MH | micro-hydro |
| $H_i$ | output of hidden layer | BM | biomass |
| $F_i$ | feedback weight of consequent part | SC | super capacitor |
| $\beta_{ij}$ | output of consequent part | UG | utility grid |
| $\gamma$ | cost function | CS | charging station |
| $\theta_i$ | recurrent weight of antecedent part | PHEV | plug-in hybrid-electric vehicle |
| **PV Cell Parameters** | | SAM | standard additive model |
| $q$ | charge of electron, $1.602x10^{-19}$C | FsNN | fourier series neural network |
| $A$ | identifier factor of pn-junction (between 1 and 5) | MHW | mexican hat wavelet |
| $k$ | Boltzman's constant, $1.380658x10^{-23}$J/K | CW | chebyshev wavelet |
| $k_i$ | cell's short circuit current temperature coefficient | WF | wavelet function |
| $R_s$ | cell's series resistance | SAM | standard additive model |
| $R_p$ | cell's parallel resistance | PMD | Pakistan meteorological department |
| | | DHA | Defense housing authority |
| | | ISE | integral square error |
| | | ISTE | integral time square error |
| | | IAE | integral absolute error |
| | | ITAE | integral time absolute error |
| | | THD | total harmonic distortion |
